# Identification of SanA as a novel regulator of peptidoglycan biogenesis in *Escherichia coli*

**DOI:** 10.1371/journal.pgen.1011712

**Published:** 2025-05-22

**Authors:** Bhargavi Gundavarapu, Krishna Chaitanya Nallamotu, Vishnu Vachana Murapaka, Balaji Venkataraman, Lutikurti Saisree, Manjula Reddy

**Affiliations:** 1 CSIR-Centre for Cellular and Molecular Biology, Hyderabad, India; 2 Academy of Scientific and Innovative Research (AcSIR), Ghaziabad, India; Norwegian University of Life Sciences: Norges miljo- og biovitenskapelige universitet, NORWAY

## Abstract

Gram-negative bacterial cell envelope consists of a surface-exposed lipid bilayer (outer membrane or OM) that serves as a permeability barrier to maintain the cellular integrity. Beneath the OM is the periplasmic space that harbours peptidoglycan (PG), a highly cross-linked mesh-like glycan polymer closely encasing the inner membrane (IM). During growth of a bacterium balanced synthesis of the envelope components is required to maintain the cellular integrity, of which little is known. In this study, we identify *sanA*, an ORF of unknown function encoding a predicted IM-anchored protein as a factor contributing to balanced synthesis of PG in *E. coli*. Absence of SanA increased the rate of nascent PG strand incorporation, and restored growth and viability to several mutants defective in either cell division or cell elongation. Detailed mutant analysis of *sanA* showed that it is defective in the envelope barrier properties. Interestingly, overexpression of the periplasmic endopeptidases that cleave the cross-links of the PG mesh was able to alleviate the phenotypes of *sanA* mutant implying the envelope defects are due to alterations in the PG sacculus. Additionally, a SanA variant (^SS^DsbA-SanA) targeted to the periplasm, complemented the SanA^−^ phenotypes suggesting it functions in the periplasmic phase of the PG synthesis. Further, we find that SanA functions independently of its paralog, ElyC, known to regulate the synthesis of enterobacterial common antigen (ECA), a surface polysaccharide found in the cell envelopes of most enteric bacteria. Overall, our results suggest a role for SanA in the maintenance of optimal PG synthesis, providing evidence for the existence of an additional layer of regulation in Gram-negative cell envelope biogenesis.

## Author summary

Gram-negative bacteria are surrounded by a cell envelope made up of two lipid-rich membranes and a mesh-like peptidoglycan (PG) sacculus sandwiched between them. Together, the cell envelope forms a formidable barrier against the entry of large hydrophobic molecules to protect cells from environmental stresses. During growth of a bacterium, balanced synthesis of the envelope components is required to maintain the cellular integrity; however, factors involved in regulation of synthesis of the envelope components are not completely understood. Using extensive genetic and molecular analyses, here, we show that *sanA*, a gene of unknown function in a model bacterial system, *Escherichia coli*, contributes to controlled synthesis of PG. Given the essentiality of the cell envelope, this study furthers our understanding of its biogenesis and aids in the identification of novel drug targets.

## Introduction

The Gram-negative bacterial cell envelope is a complex multi-layered structure that makes a formidable barrier shielding cells against various environmental insults and stress conditions [1]. It has a surface-exposed outer membrane (OM) and an inner membrane (IM) lining the cytoplasm. OM is an asymmetric lipid bilayer consisting of lipopolysaccharides (LPS) in the outer leaflet and phospholipids (PL) in the inner leaflet whereas the IM is a symmetric bilayer comprising PLs in both leaflets. Additionally, OM is decorated with various surface-exposed polysaccharides such as O-antigen and Enterobacterial Common Antigen (ECA), which is found exclusively in members of *Enterobacterales* [1,2]. Between these two membranes is the periplasmic space that harbours a covalently closed, net-like macromolecule, the peptidoglycan (PG), which provides mechanical strength and shape to the cell [3–6].

Of the envelope constituents, LPS is essential to maintain the permeability barrier function of the OM. It is made up of lipid A, a hexa-acylated glucosamine disaccharide linked to O-antigen via a core oligosaccharide. Biosynthesis of lipid A is initiated in the cytosol by fatty acylation of a sugar-nucleotide, UDP-*N*-acetylglucosamine (UDP-GlcNAc). Subsequent steps lead to the formation of hexa-acylated Kdo_2_-lipid A, which is then flipped across the IM into the periplasm before being assembled to form mature LPS and transported into the outer leaflet of OM by Lpt machinery [7,8].

In addition, the surface of the OM in certain classes of Gram-negative bacteria is adorned with complex polysaccharide antigens such as ECA and O-antigen. ECA is a polymer of simple repeating trisaccharide units whereas O-antigen is a complex sugar polymer composed of multiple oligosaccharide units [2,9,10]. The synthesis of both ECA and O-antigen begins with transfer of a GlcNAc-1-phosphate moiety from UDP-GlcNAc to an IM-lipid carrier, undecaprenyl phosphate (UndP) by the enzyme WecA to produce Lipid-I^ECA/O^. Subsequently, Lipid-I^ECA/O^ is committed either towards O-antigen synthesis by the action of WbbL or towards ECA synthesis by WecG. However, in *E. coli* K12 strains, the O-antigen synthesis is lost due to an IS5 insertion mutation in the *wbbL* gene [2,9,10].

**Fig 1:**
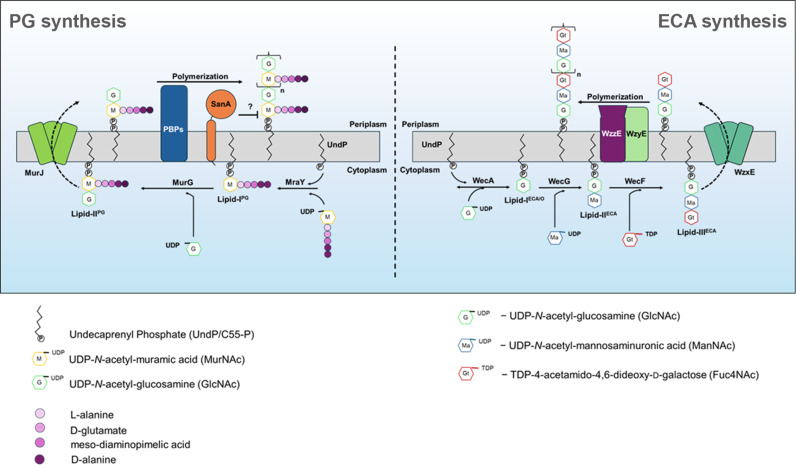
Schematic representation of the peptidoglycan (PG) and Enterobacterial common antigen (ECA) biosynthetic pathways. PG synthesis occurs in two distinct cellular compartments. In the cytoplasm, UndPP-MurNAc (pentapeptide)-GlcNAc (Lipid-II^PG^) moieties are synthesized, flipped across the IM into the periplasmic space by MurJ flippase, and subsequently polymerized into the growing sacculus by the activity of PG synthases (PBPs). Herein, our study provides evidence for SanA, a protein of unknown function to be a novel regulator of PG biosynthesis, likely controlling the step of nascent PG formation in the periplasm. In parallel, the biosynthesis of ECA begins in the cytoplasm wherein a series of enzymatic reactions leads to the formation of Lipid-III^ECA^ (UndPP-GlcNAc-ManNAc-Fuc4NAc) which is then translocated across the IM into the periplasm by the flippase WzxE. In the periplasm, the three-sugar unit is polymerized through WzzE-WzyE complex to generate the mature ECA polymer.

In the ECA biosynthesis pathway, the committed step is formation of Lipid-II^ECA^ from Lipid-I^ECA/O^ by WecG, which is then followed by formation of Lipid-III^ECA^ by WecF. Further, Lipid-III^ECA^ is translocated into the periplasm by WzxE flippase and polymerized by WzyE. The number of repeat units in the polymer is controlled by the co-polymerase, WzzE (Fig 1). The ECA polymer is then either ligated to an LPS moiety by WaaL ligase to produce ECA_LPS_, ligated to phosphatidylglycerol to make ECA_PG,_ or circularized to form soluble periplasmic ECA_cyc_. ECA_LPS_ and ECA_PG_ are surface-exposed, whereas ECA_cyc_ remains in the periplasmic space [11].

In parallel, PG biosynthesis begins in the cytosol with the conversion of UDP-GlcNAc to UDP-MurNAc by MurA and B, marking the first committed step in this pathway (Fig 1). Next, the sequential action of MurC, -D, -E, and -F ligases leads to the formation of UDP-MurNAc-pentapeptide which is then attached to the lipid carrier, UndP by MraY to form Lipid-I^PG^ [12]. MurG then attaches a UDP-GlcNAc moiety to MurNAc-pentapeptide of Lipid-I^PG^, to make Lipid-II^PG^ [UndPP-MurNAc (pentapeptide)-GlcNAc] which is then flipped into the periplasm by MurJ flippase. Here, the Lipid-II precursors are polymerized to form PG mesh for either sidewall synthesis during cell elongation or septal synthesis during cell division by dedicated PG biosynthetic machineries [3–6]. As PG forms a continuous network around the IM, its expansion requires hydrolysis of the pre-existing cross-links for the incorporation of new material by the activity of redundant cross-link specific endopeptidases, MepS, -M and -H [13,14].

PG polymerization in the periplasm involves two consecutive enzymatic reactions: a glycosyltransferase activity for polymerizing the disaccharide sugars to make the glycans followed by a transpeptidation reaction that forms cross-links between the peptides of adjacent glycan strands, eventually leading to the formation of a mesh-like sacculus. During sidewall synthesis, RodA-PBP2, a glycosyltransferase-transpeptidase pair forms a scaffold whereas the gaps in the scaffold are filled by two bifunctional wall synthases, PBP1A and PBP1B. On the other hand, the divisomal glycosyltransferase-transpeptidase pair, FtsW-FtsI, synthesizes the septal PG at the division site. The division process is initiated by formation of a septal ring by FtsZ, a cytoskeletal protein which then recruits other divisomal components such as FtsA, FtsEX, FtsK, FtsQ, FtsL, FtsB, FtsW, FtsI, FtsN, and FtsP to facilitate the wall formation. Subsequently, the division-specific amidases (AmiA, -B, and -C) split the septal PG leading to the separation of two daughter cells [5,15].

During growth, balanced synthesis of the envelope is crucial to maintain the cellular integrity, requiring stringent regulation of each of the components. In this study, we report that *sanA*, an ORF of unknown function encoding a DUF218-domain containing protein contributes to the regulation of PG synthesis in *E. coli*. We identified *sanA* in a genetic screen as its deletion suppressed the growth defects of several cell division or cell elongation mutants. Further, we observed that absence of *sanA* leads to a high level of nascent PG strand incorporation into the sacculi. Extensive analysis of *sanA* deletion mutants showed that these were sensitive to low-osmolarity conditions, treatment with SDS+EDTA or vancomycin indicating compromised cell envelope integrity. Interestingly, we find that overexpression of cross-link specific PG endopeptidases such as MepH, MepS or MepM which open the PG mesh abrogates the envelope defects of *sanA* mutant. Moreover, a periplasmic *sanA* variant (^SS^DsbA-SanA) lacking the IM anchor was functional, showing SanA works in the periplasm, probably at the step of PG polymerization. Furthermore, a set of three conserved amino acid residues forming a potential catalytic triad was found to be critical for its function implying an enzymatic activity to SanA. Altogether, these findings show that absence of *sanA* leads to dysregulation of PG formation suggesting a role for it in PG synthesis.

Interestingly, it has been shown earlier that ElyC, a paralog of SanA containing a DUF218 domain, regulates ECA synthesis in *E. coli* with *elyC* deletion mutants showing decreased PG and increased levels of ECA [16,17]. In this context, we find that SanA works independently of ECA synthesis as well as ElyC. Taking together the earlier studies on ElyC [16,17], and the findings reported in this study, we propose that SanA and ElyC function in parallel to balance the levels of PG and ECA, respectively, as these pathways share common precursors for their synthesis, thereby ensuring a proper growth of cell envelope in *E. coli*.

## Results

### Absence of *sanA* restores growth to a mutant defective in FtsI, a division-specific D, D-transpeptidase

**Fig 2:**
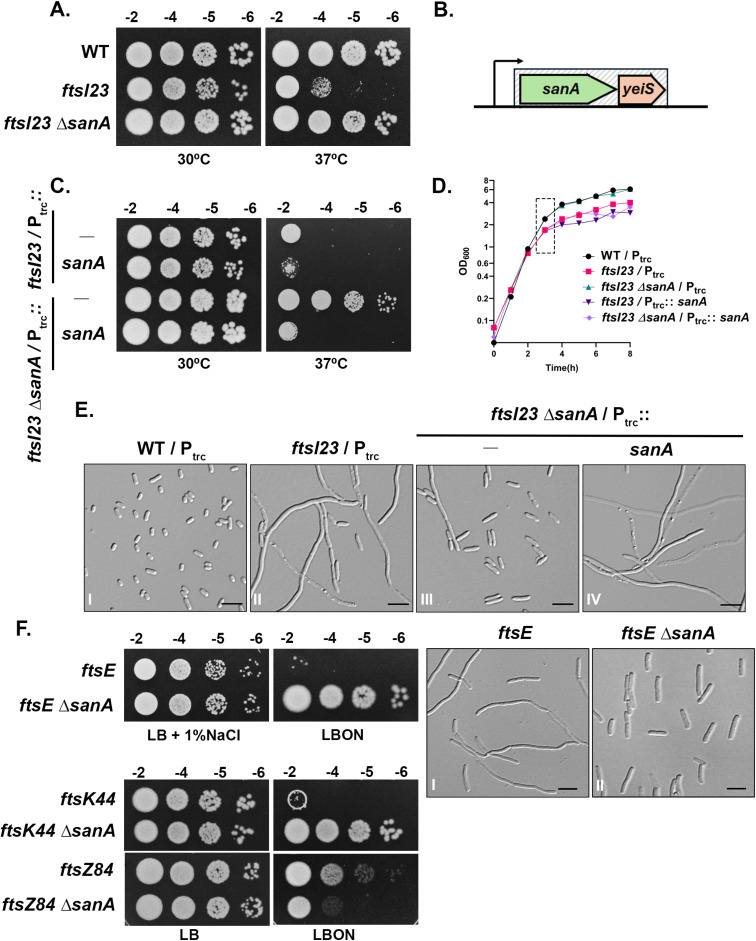
Absence of *sanA* rescues the growth defects of several cell division mutants. (A) Overnight cultures of WT, *ftsI23*, and *ftsI23 ∆sanA* strains were serially diluted and 4 μL of each dilution were spotted on LB plates and incubated at indicated temperatures. Growth phenotypes were scored after 20-24 h incubation. (B) Operonic arrangement of *sanA* and *yeiS* genes located at 48.11-48.12 min on the *E. coli* chromosome (not drawn to scale). Arrow indicates the direction of transcription. (C) Indicated strains were grown in LB with ampicillin at 30°C and tested for viability as described above on LB plates supplemented with ampicillin and 10 µM IPTG. (D, E) Overnight cultures of WT or its derivatives carrying pTrc99a or pTrc99a-*sanA* were sub-cultured at 1:100 dilution in LB supplemented with ampicillin and 10 µM IPTG at 37°C. Growth was monitored at regular intervals. Cells were collected after 3 h for visualization using Differential Interference Contrast (DIC) microscopy. Scale bars represent 5 µm. (F) *ftsE* and *ftsE ∆sanA* strains were grown overnight in LB with 1% NaCl at 30°C and cell viability was tested on indicated plates at 30°C. Microscopy was done as described above. Overnight cultures of strains carrying *ftsK44*, or *ftsZ84* alleles were grown at 30°C, serially diluted and viability was assessed on indicated plates (LB at 30°C and LBON at 37°C).

During a genetic screen performed to obtain factors that influence cell division (as described in Materials and Methods), we observed that absence of *sanA*, a gene of unknown function, restored growth to a strain carrying a temperature-sensitive mutation in *ftsI* (*ftsI23*) [18]. In addition to re-establishing growth at the restrictive temperature, *sanA* deletion suppressed the division defects of the *ftsI23* mutant (Fig 2A–2E). In *E. coli*, *sanA* is the first gene in a two-gene operon (*sanA*-*yeiS*) located approximately at 48 min on its chromosome (Fig 2B). However, deletion of the downstream gene, *yeiS,* did not affect the growth phenotypes of the *ftsI23* mutant (S1A Fig). Furthermore, a plasmid encoding *sanA* cloned downstream to an IPTG-inducible promoter (P_trc_::*sanA*) completely abrogated the suppression conferred by the deletion of *sanA* in the *ftsI23* mutant (Fig 2C–2E).

### Deletion of *sanA* restores growth and division to several other mutants defective in cell division

To examine the effect of SanA on the phenotypes of other division mutants, *sanA* deletion was introduced into multiple strains each carrying mutations in *ftsZ* (*ftsZ84*), *ftsA* (*ftsA12*), *ftsE* (*ftsE*::Tn*10*dCm), *ftsX* (*ftsX*::Tn*10*dCm), *ftsK* (*ftsK44*), *ftsQ* (*ftsQ1*) or *ftsP* (Δ*ftsP*::Kan) [19,20]. Interestingly, deletion of *sanA* effectively suppressed the growth defects of all the above mutant strains except that of the strains carrying mutations in the early division genes, *ftsZ (ftsZ84*) or *ftsA* (*ftsA12*) (Figs 2F, and S1B,S1C). In addition, absence of SanA had no significant effect on the growth of a triple amidase (Δ*amiABC*) mutant defective in the process of cell separation (S1D Fig). Overall, the above results show that absence of *sanA* is able to overcome the growth defects of several cell division mutants.

### Absence of SanA rescues the growth defects of mutants lacking other PG synthases

**Fig 3:**
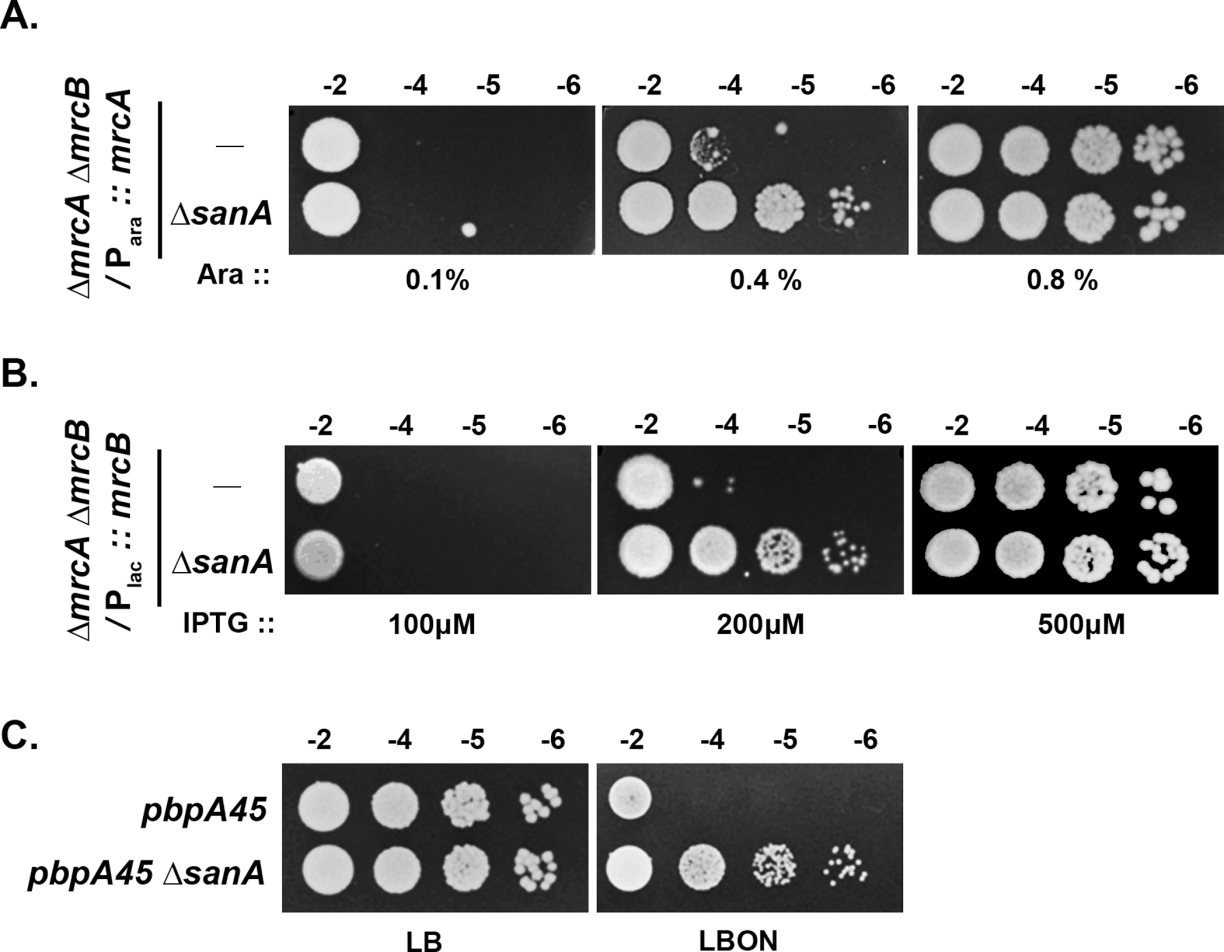
Deletion of *sanA* suppresses the growth defects of cell elongation mutants. (A) A double mutant of ∆*mrcA* ∆*mrcB* carrying a chromosomal copy of *mrcA* downstream to an arabinose-inducible promoter (P_ara_::*mrcA*) and its *sanA* deletion derivative were grown overnight with 0.5% arabinose, serially diluted and tested for viability on LB plates supplemented with arabinose (Ara) at 37°C. (B) Viability of a double mutant of ∆*mrcA* ∆*mrcB* carrying a chromosomal copy of *mrcB* downstream to an IPTG-inducible promoter (P_lac_::*mrcB*) or its *sanA* derivative was tested as described above. IPTG was used at indicated concentrations. (C) Viability of strains carrying *pbpA45* or *pbpA45 ∆sanA* mutations grown overnight in LB at 30°C was tested on indicated plates (LB at 30°C and LBON at 42°C).

As *sanA* deletion suppressed division-defective PG synthase mutation (*ftsI23*), we examined its effect on the phenotypes of other PG synthase mutants. Fig 3 shows that absence of SanA is able to rescue the growth of strains depleted of either PBP1A (encoded by *mrcA*) or PBP1B (encoded by *mrcB*), the bifunctional aPBPs that facilitate wall synthesis during PG expansion (using a Δ*mrcA* Δ*mrcB* double deletion strain carrying either P_ara_::*mrcA* or P_lac_::*mrcB* as shelter) [21]. In addition, growth of a mutant carrying *pbpA45*, a hypomorphic allele of PBP2 [22], a monofunctional transpeptidase involved in scaffold-synthesis, was enhanced in absence of *sanA* (Fig 3C). However, *sanA* deletion did not confer any growth advantage to strains defective in the synthesis of other envelope components such as LPS or PL (S2A and S2B Fig). Overall, this data suggested that SanA may have a function in PG-associated processes.

### Absence of SanA increases nascent PG synthesis

**Fig 4:**
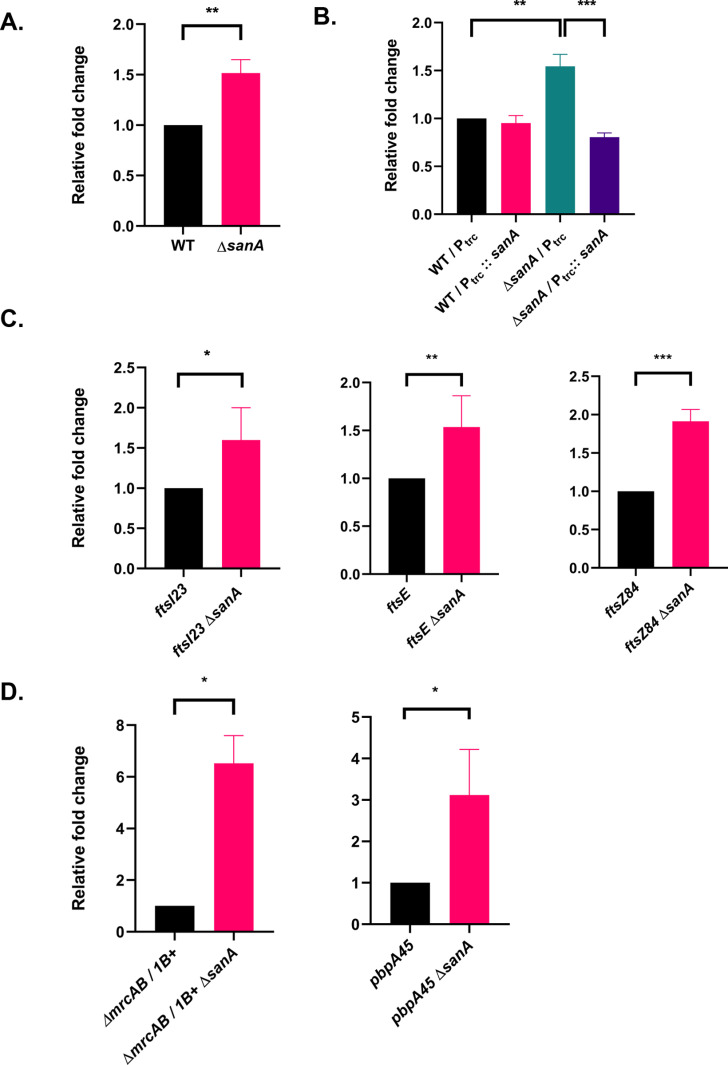
Effect of SanA deletion on nascent peptidoglycan (PG) synthesis. Nascent PG strand incorporation into the sacculi was measured using ^3^H-mDAP as described earlier (23). All strains had a deletion of *lysA* to prevent mDAP conversion to lysine and grown in Min-A (minimal-A medium) supplemented with 0.2% glucose and 0.5% Casamino acids. PG synthesis was measured in normalised cultures that were grown to an OD_600_ value of 0.4-0.6. (A) WT and *sanA* mutant strains are grown at 37°C and PG synthesis measured as described above. (B) Above strains carrying either pTrc99a or pTrc99a-*sanA* were grown at 37°C with 10 µM IPTG and the ^3^H-mDAP incorporation assay was carried out as described above. (C) Cultures of *ftsI23*, *ftsE*, and *ftsZ84*, along with their *sanA* deletion derivatives, were grown at 30°C and processed for ^3^H-mDAP incorporation as described earlier. (D) Derivatives of ∆*mrcA* ∆*mrcB* with P_lac_:: *mrcB* (∆*mrcAB*/*1B+*) strain with or without *sanA* deletion were grown at 37°C in presence of 100 µM IPTG and ^3^H-mDAP incorporation assay was performed as described above. *pbpA45* and *pbpA45 ∆sanA* were grown at 30°C for ^3^H-mDAP incorporation assays. All the measurements were done at least three times and ratios were calculated and plotted as bar graphs.

As above results suggested a role for SanA in PG metabolism, we examined the composition of PG sacculi in WT and *sanA* deletion mutant. However, we did not find any significant alteration in PG composition of these strains (S2C and S2D Fig). Next, we examined the rate of nascent PG strand incorporation by measuring PG synthesis using tritiated meso-diaminopimelic acid (^3^H-mDAP) as described in Materials and Methods [23]. Surprisingly, *sanA* deletion showed a significant increase (nearly 1.5 to 2-fold) in the rate of PG incorporation compared to that of WT (Fig 4A), which is complemented by a plasmid-borne copy of *sanA* (Fig 4B). We also examined the rate of PG synthesis in both division and elongation mutants (carrying *ftsI23*, *ftsE*, *ftsZ84*, Δ*mrcA* Δ*mrcB*/ P_lac_::*mrcB*, or *pbpA45* alleles) using ^3^H-mDAP incorporation assay. Deletion of *sanA* increased mDAP counts in all the strains tested above (Fig 4C and 4D) like that of WT. Interestingly, *sanA* deletion increased the mDAP incorporation even in the strain carrying *ftsZ84*, in which the growth rescue was not observed. In summary, *sanA* deletion increased the nascent PG synthesis in all the strains tested, implying its basis of suppression is through a generalized increase of PG. Importantly, these results show that SanA negatively regulates PG synthesis likely contributing to balanced formation of PG in *E. coli*.

**Fig 5:**
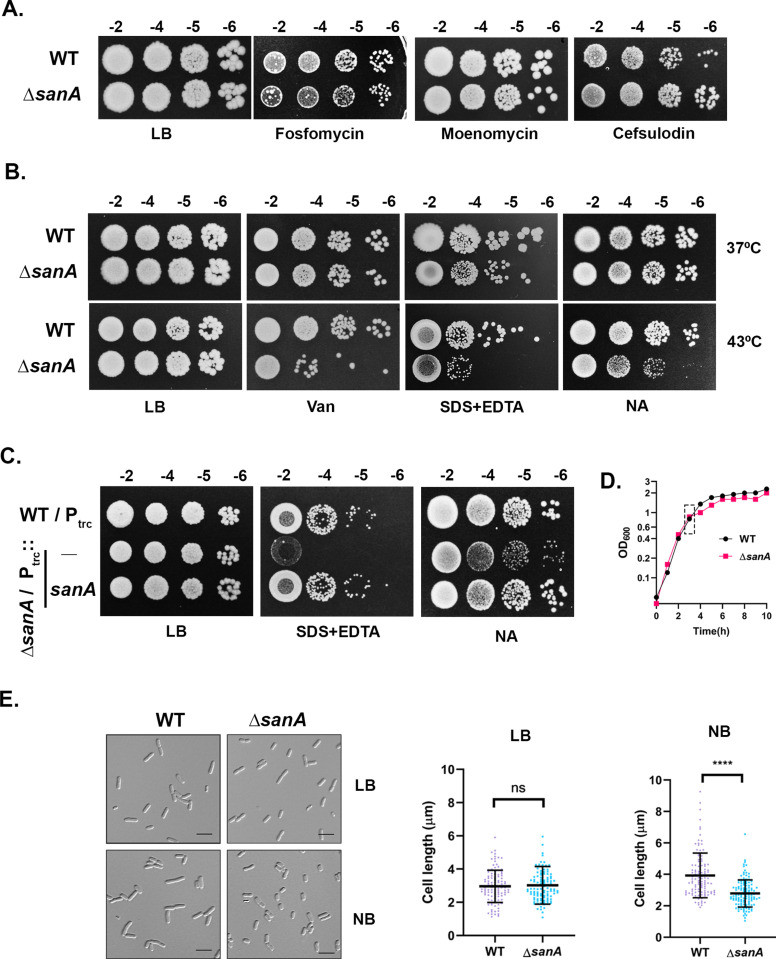
Phenotypes of *sanA* deletion mutant. (A) Effect of cell-wall targeting antibiotics was examined on the growth of WT and ∆*sanA* strains on LB, LB+fosfomycin (1 µg/mL), LB+moenomycin (6 µg/mL), or LB+cefsulodin (24 µg/mL) at 37°C. (B) Viability assays of WT and ∆*sanA* strains were done on LB, LB + 200 µg/mL vancomycin (Van), LB + 1.0% SDS + 0.5 mM EDTA, or NA plates at indicated temperatures. (C) Growth of indicated strains was scored at 43°C on LB + 1.0% SDS + 0.5 mM EDTA and NA plates supplemented with 10 µM IPTG. (D, E) Overnight cultures of WT and ∆*sanA* were diluted 1:100 in Nutrient Broth (NB) and LB and grown at 43°C. Growth was monitored at regular intervals. At OD_600_ of 0.6-0.8, cells were collected, immobilized on 1% agarose pads, and visualized using DIC microscopy. For cell length measurements, approximately 100 cells were used and data were analysed by ImageJ software.

As the above results suggested a role for SanA in cell wall synthesis, we evaluated the effect of various cell wall-targeting antibiotics on the growth of *sanA* mutant (Fig 5A). Here, we observed that treatment with fosfomycin, which inhibits the formation of cytosolic PG precursors by targeting MurA, or moenomycin, which targets the glycosyltransferase activity of class A PBPs had no discernible effect on the growth of *sanA* mutant. However, interestingly, cefsulodin, a β-lactam antibiotic which targets the transpeptidase activity of class A PBPs conferred a moderate growth advantage compared to that of WT supporting a role for SanA in PG synthesis.

### Absence of *sanA* confers cell envelope defects

Considering an earlier report that *sanA* mutants exhibit permeability defects at elevated temperatures (43°C), we revisited this phenotype. Consistent with previous findings [24], we observed that the *sanA* mutant is indeed sensitive to vancomycin and treatment with SDS+EDTA at higher temperatures (Fig 5B and 5C). In addition, we found that the mutant exhibits reduced growth rate in low-osmolarity media such as Nutrient Agar (NA) which is rescued by addition of osmolytes such as NaCl or sorbitol (Figs 5B and S3A). However, all these phenotypes were evident only when cells were grown at 43°C but not at 37°C, as reported earlier [24]. To check whether the permeability defects are arising because of LPS deficiency, we examined its levels, and consistent with the earlier study [24], no alterations in LPS were observed (S3B Fig). While no obvious morphological defects were observed in LB, *sanA* mutants grown in NB (Nutrient Broth) were significantly smaller compared to that of WT (Figs 5D, 5E and S3C). In summary, the above experiments show that absence of SanA leads to compromised envelope barrier properties which are LPS-independent.

### Interactions of *sanA* with ECA biosynthetic pathway

**Fig 6:**
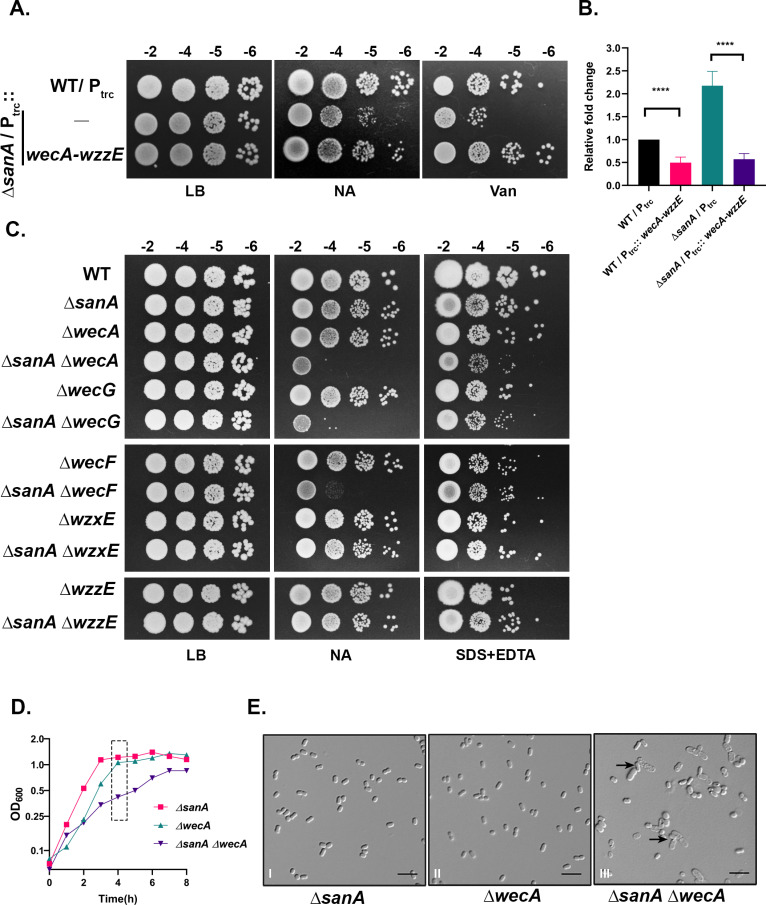
Genetic interactions of *sanA* with ECA pathway. (A) Indicated strains were grown overnight in LB containing ampicillin and growth was checked on LB, NA, and LB + 200 μg/mL vancomycin plates supplemented with ampicillin and 10 µM IPTG. (B) The above-described strains (with additional *lysA* mutation) were used to measure ^3^H-mDAP incorporation as described earlier. Cells were grown with 10 µM IPTG. (C) Growth of the indicated strains was examined on LB, LB + 1.0% SDS + 0.5 mM EDTA or NA plates incubated at 37°C. (D, E) Growth of *sanA*, *wecA* and *sanA wecA* mutant strains was tested in NB at 43°C by measuring OD_600_ at regular intervals and after 4h of growth, cells were collected, immobilized on 1% agarose pads, and visualized using DIC microscopy. Black arrows indicate cell lysis. The scale bar represents 5 μm.

To investigate the underlying basis of the *sanA* mutant phenotypes, we performed a genetic screen using a multicopy plasmid library (as described in Materials and Methods), selecting colonies that grew better on NA plates at 43°C. Among the suppressors identified, a clone carrying the region encompassing both *wecA* and *wzzE* genes (early genes in the operon encoding ECA biosynthetic pathway) conferred a moderate growth advantage to *sanA* mutant (S4A Fig). We confirmed the suppression by constructing a clone carrying *wecA-wzzE* genes on a medium-copy plasmid vector downstream to an IPTG-inducible promoter (P_trc_::*wecA-wzzE*) (Fig 6A). To understand the basis of suppression, we measured the rate of PG synthesis in strains overexpressing *wecA-wzzE* genes. Interestingly, overexpression of these genes decreased the incorporation of ^3^H-mDAP to two-fold both in the WT and *sanA* mutant, suggesting that *wecA-wzzE* overexpression generally reduces PG synthesis (Fig 6B) most likely by channelling the precursors away from PG into ECA pathway.

We next examined the *sanA* mutant phenotypes in absence of ECA by introducing deletions in each of the genes involved in ECA synthesis into a *sanA* deletion strain. Here, we observed that deletion of *wecA*, *wecG* or *wecF*, the early genes in ECA biosynthesis in *sanA* mutant resulted in synthetic sickness on NA plates at 37 °C wherein the single mutants had no discernible defects (Fig 6C). The double deletion mutants also exhibited cell lysis in late exponential phase (Fig 6D and 6E). These observations suggested the additive sickness could be due to exacerbation of *sanA*’s defects in absence of ECA implying SanA functions outside of ECA pathway. In support of this, strains carrying deletion of *wzxE* or *wzzE*, the genes encoding the flippase or the co-polymerase of the ECA pathway which still make functional ECA due to the presence of redundant flippase/ polymerase (of O-antigen pathway) did not confer additive sickness (Fig 6C) [11].

### SanA and ElyC work independently in PG and ECA pathways

**Fig 7:**
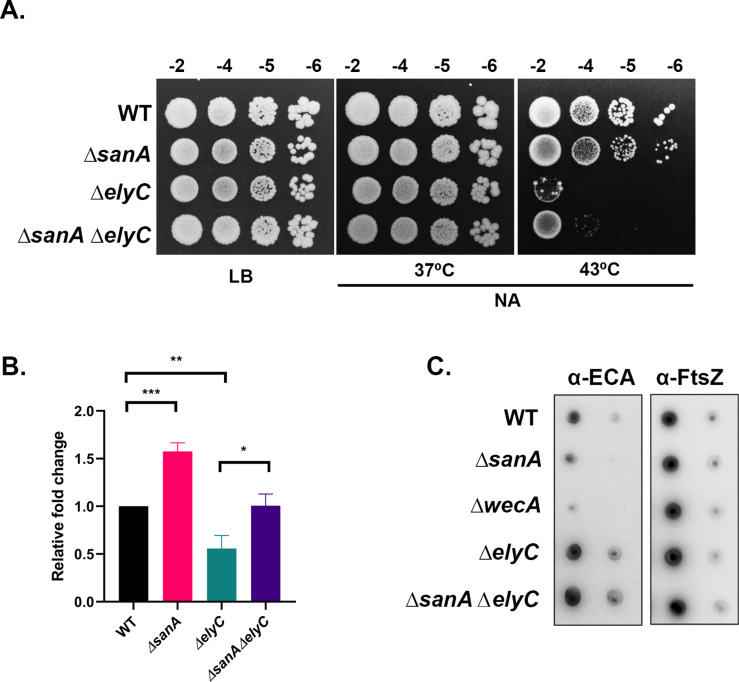
Genetic interactions of SanA with ElyC. (A) Viability of the indicated strains was tested on LB and NA plates at indicated temperatures. (B) Above strains carrying *lysA* deletion were used to estimate nascent PG synthesis using ^3^H-mDAP incorporation. (C) Overnight cultures of the indicated strains were diluted 1:100 in LB and grown at 43°C to an OD_600_ of 2.0. Cells were collected and processed for dot blot analysis as described in Materials and Methods. Δ*wecA* mutant is used as a negative control for detection of ECA and FtsZ as a normalization control.

Interestingly, ElyC, a paralog of SanA is shown to regulate ECA synthesis with *elyC* deletion mutants exhibiting elevated ECA levels and decreased PG synthesis [16,17]. To examine whether ElyC and SanA are functionally related to each other, we constructed *sanA elyC* double mutants and assessed their phenotypes. However, we find *elyC* single mutants themselves do not grow on NA plates (NA^S^) at 43°C (Fig 7A). We then measured the rate of ^3^H-mDAP incorporation in these strains. In agreement with a previous study, approximately 50% less mDAP incorporation was observed in *elyC* single mutant [16]. More importantly, absence of *sanA* was able to elevate the mDAP counts approximately 1.5- to 2- fold in *elyC* mutant background as well (Fig 7B), suggesting the role of SanA in PG synthesis is independent of ElyC. Further, we measured ECA levels in these mutants using anti-ECA antibodies in a dot blot assay and find that the elevated ECA levels observed in *elyC* single mutant were not affected by absence of SanA (Fig 7C). Overall, these results suggest that the two paralogs, SanA and ElyC play distinct roles that are not interdependent on each other.

### Cleavage of PG mesh alleviates SanA^−^ phenotypes

**Fig 8:**
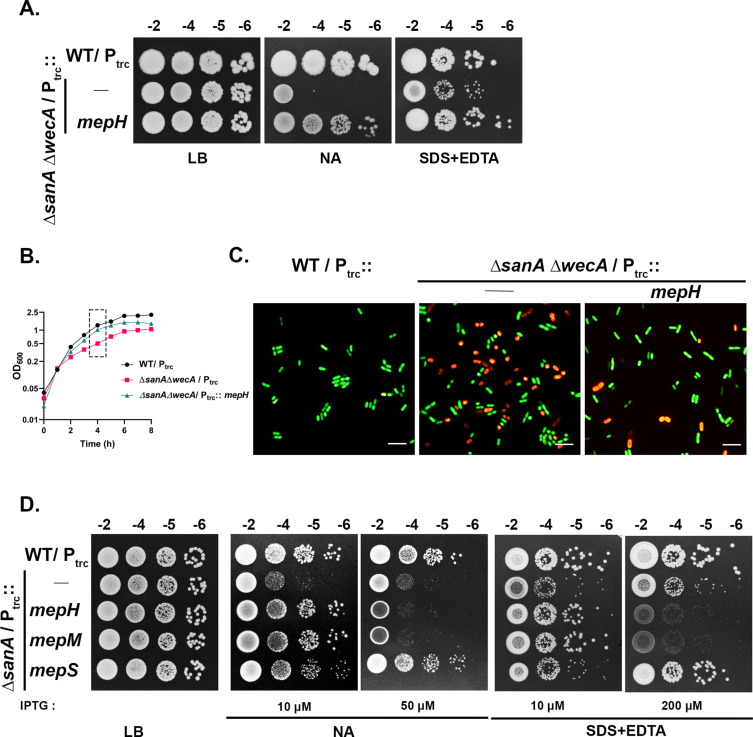
Overexpression of PG cross-link cleaving hydrolases alleviates *SanA*^−^ phenotypes. (A) Indicated strains were grown overnight in LB+ampicillin and growth was examined on LB, NA, LB + 1.0% SDS + 0.5 mM EDTA plates at 37°C. IPTG was used at 50 μM. (B, C) Overnight cultures of WT and its derivatives were diluted 1:100 in NB containing ampicillin and grown at 43°C. Growth was monitored at regular intervals, cells were collected after 4h of growth, and viability was tested using live-dead staining as described in Materials and Methods. Green and red indicate live and dead cells, respectively. (D) Strains were grown overnight in LB and viability was tested on LB, NA, LB + 1.0% SDS + 0.5 mM EDTA plates supplemented with indicated concentrations of IPTG at 43°C.

To further dissect the function of SanA, we performed another multicopy suppressor screen (using the pACYC plasmid library) utilizing the severe NA-sensitivity phenotype of *sanA wecA* double deletion strain at 37°C. Among the suppressor plasmids, a clone containing a region encompassing *grxD-mepH* genes of the *E. coli* chromosome rescued both the NA- and SDS+EDTA-sensitivities of *sanA* mutant (S4B Fig). Subsequently, a plasmid carrying *mepH* alone (P_trc_::*mepH*) [13] was found to restore the barrier defects of both *sanA wecA* and *sanA* mutants (Fig 8A–8D). MepH is a periplasmic PG endopeptidase that cleaves the peptide cross-links between the glycan strands to open up the mesh during expansion of the PG sacculus [13,25]. This observation prompted us to examine whether other cross-link specific PG endopeptidases were able to rescue the SanA^−^ phenotypes. Indeed, overexpression of either MepS or MepM, the two major cross-link cleaving PG endopeptidases [13], restored the barrier defects of *sanA* mutant suggesting cleaving the PG mesh is beneficial to this mutant (Fig 8D). Importantly, this observation indicated that the PG sacculus in absence of *sanA* could be aberrant leading to barrier defects which are alleviated by the cleavage of cross-links.

### Evidence for a catalytic role of SanA in the periplasm

**Fig 9:**
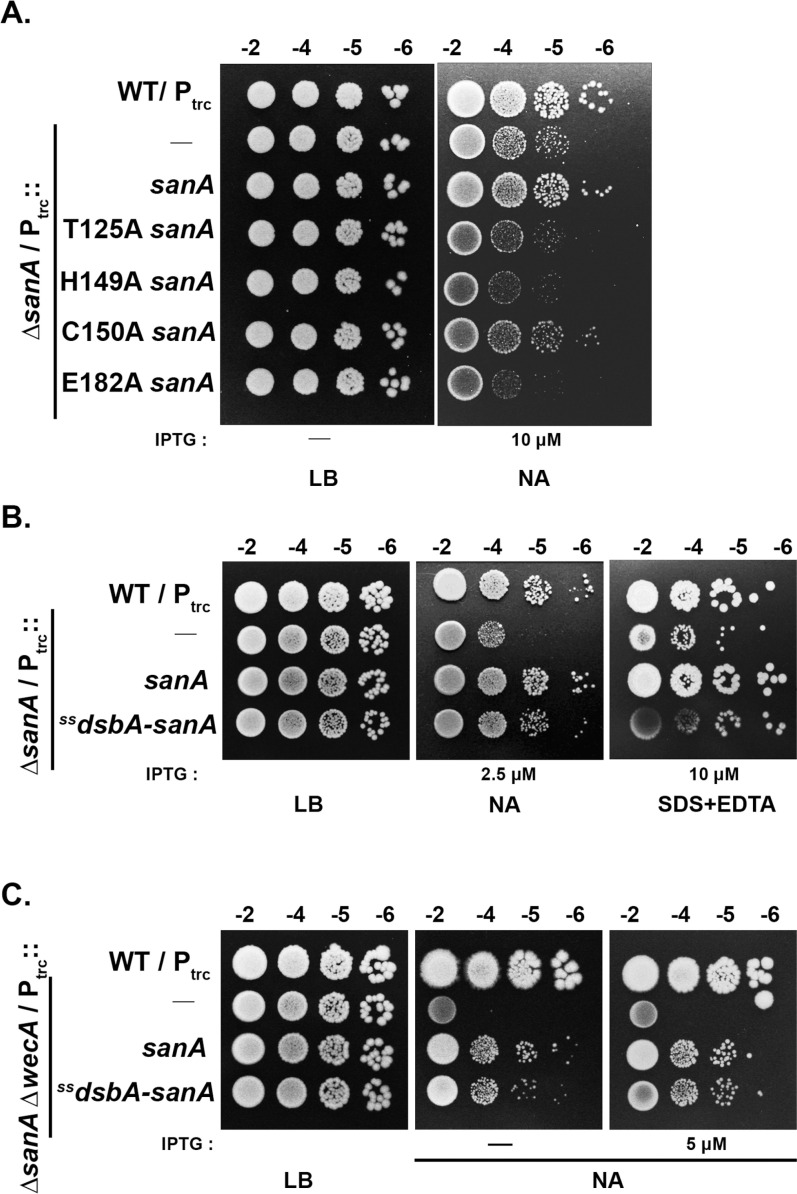
Evidence for a catalytic role for SanA in periplasm. (A) Complementation of SanA^−^ phenotypes by plasmid-borne *sanA* variants (T125A; H149A; C150A; E182A) was examined by assessing growth of indicated strains on LB or NA plates at 43°C. C150A variant was able to complement the San^−^ phenotype whereas the other variants did not. (B) Complementation of SanA^−^ phenotypes by a plasmid-borne ^ss^*dsbA-sanA* variant was tested as described above on indicated plates at 43°C. (C) Complementation of NA^S^ of *sanA wecA* double mutant by the ^ss^*dsbA-sanA* variant was tested as described above on indicated plates at 37°C.

*E. coli* encodes four DUF218 domain containing proteins: SanA, ElyC, YdcF, and YgjQ. Among these, SanA, ElyC, and YgjQ are predicted to be IM-anchored with their DUF218 domains exposed to the periplasm, whereas YdcF is predicted to be a cytosolic enzyme involved in anaerobic metabolism [16,26]. In an attempt to understand whether DUF218 domain has any catalytic function, we analysed the structural and sequence alignment of these paralogs, and identified a conserved triad of threonine, histidine, and glutamate, which likely forms a catalytic core in these proteins (S5A Fig). To evaluate the functional importance of these conserved residues, we generated alanine substitution variants of SanA and assessed their ability to complement the *sanA* mutant. None of the three variants were able to complement the SanA^−^ phenotype, despite being expressed at levels comparable to the wild-type protein (Figs 9A and S5B). These findings suggest that the conserved triad residues are critical for its function, implying an enzymatic activity for SanA.

To examine whether SanA works in the periplasm, we replaced its membrane anchor with the signal sequence from DsbA (^SS^*dsbA*-*sanA*) and examined its functionality. As shown (Fig 9B and 9C), this variant complemented the SanA^−^ phenotypes confirming its site of action is in the periplasm. Overall, these results suggest that SanA functions in the periplasmic stage of PG synthesis, likely at the step of PG polymerization.

## Discussion

Biogenesis of the cell envelope must be coordinated with the cellular growth, therefore necessitating a tight regulation of the biosynthetic pathways of envelope components. Dysregulation of these processes may compromise the envelope integrity, potentially leading to cell death. Herein, we provide evidence for the existence of an additional layer of regulation in PG biogenesis of *E. coli* by identifying SanA, a protein of unknown function as a factor contributing to the balanced synthesis of PG. Interestingly, ElyC, a paralog of SanA, was earlier shown to regulate ECA, an envelope-associated glycopolymer conserved in enteric bacteria. Taking together the earlier findings on ElyC and our own, we propose that SanA and ElyC, both of which belong to the conserved DUF218-domain family of proteins, work in parallel to maintain balanced PG and ECA biogenesis in members of Enterobacteriaceae.

### Role of SanA in PG biogenesis

During our attempts to understand septal PG synthesis, we discovered that *sanA* disruption suppresses not only *ftsI23* mutant phenotypes, but also the defects of several mutants involved in cell division or elongation (Figs 2 and 3). This general suppressive ability of cell wall-associated mutants has prompted us to examine the effect of *sanA* deletion on PG synthesis. Here, we observed a significant increase in the rate of nascent PG synthesis in all the strains carrying a *sanA* mutation, suggesting SanA functions as a negative modulator of PG synthesis (Fig 4). A previous study from *Salmonella typhimurium* has shown that disruption of *sanA*^*st*^ (*sfiX*) suppressing the cell filamentation defect caused by HisH and HisF overexpression (His^c^ pleiotropic response), based on which, it was suggested that SanA has a role in PG synthesis [27].

In *E. coli*, SanA was earlier identified as a factor contributing to the resistance against vancomycin and SDS+EDTA [24]. It has also been shown to confer SDS-resistance during carbon-limited stationary-phase [28], altogether suggesting its role in the maintenance of envelope barrier properties [24,28]. Here, we find that *sanA* mutants are indeed sensitive to treatment with SDS+EDTA and vancomycin. In support of earlier observations [24], we also find that these barrier defects are not due to alterations in LPS (S3B Fig).

In an attempt to examine the basis of barrier defects in *sanA* mutant, we found that overexpression of *wecA-wzzE* is able to moderately rescue the SanA^−^ phenotypes. The overexpression was also able to reduce the rate of PG synthesis raising a possibility of decreased PG as the basis of *sanA* suppression (Fig 6A and 6B). We further tested the involvement of SanA in the ECA pathway by assessing the growth phenotypes of single and double mutants of *sanA* lacking each of the genes involved in ECA synthesis and observed that (1) the absence of ECA by itself does not lead to loss of barrier properties, and (2) a complete shutdown of ECA production is additively sick with a deletion of *sanA* likely due to a combination of dysregulated PG and loss of ECA (Fig 6C). These results allowed us to infer SanA’s role is not in ECA pathway.

Altogether, the above results showed SanA controls the formation of PG. Subsequently, identification of cross-link specific endopeptidases, MepS, -M and -H as factors suppressing the SanA^−^ phenotypes gave us a strong indication that the PG sacculus of *sanA* mutant could be dense and aberrant due to enhanced PG strand incorporation, leading to barrier defects (Fig 8A–8D). Although the basis of the suppression by these endopeptidases is not clear, it is plausible that cleavage of the peptide cross-links between the glycan strands may loosen the dense PG mesh thereby mitigating the barrier defects of SanA mutant. Moreover, the complementation of SanA^−^ phenotypes by ^ss^*dsbA*-*sanA* variant strengthens the above result indicating SanA functions at the step of nascent PG strand polymerization, most likely by controlling the flux of new subunits into the growing PG mesh.

### DUF218-family proteins contribute to Gram-negative bacterial cell envelope biogenesis

*E. coli* encodes four DUF218-domain containing proteins: SanA, ElyC, YdcF, and YgjQ. Topological predictions indicate that SanA, ElyC and YgjQ are IM-anchored with their DUF218 domains in the periplasmic space whereas YdcF is cytoplasmic [16,26]. Of these, ElyC is shown to control ECA levels, with its deletion exhibiting elevated ECA levels, particularly high ECA_PG_ [17]. Additionally, ElyC depletion leads to cell lysis at lower temperatures, accompanied with severe cell wall-related defects. This phenotype is attributed to the sequestration of the lipid carrier UndP in the ECA pathway leading to decreased PG synthesis [16].

On the other hand, SanA controls PG levels, with *sanA* mutants exhibiting elevated PG synthesis with compromised envelope permeability at high temperature. Although, dot blots revealed a moderate ECA decrease in *sanA* deletion mutant, (Fig 7C), we could not confirm through western blotting due to paucity of the antibody. Combining the above results, we propose these two DUF218 proteins work towards the maintenance of balanced PG and ECA synthesis. In support of this, both *sanA* and *elyC* genes are conserved mostly in the members of the order Enterobacterales of Gram-negative bacteria.

Among the DUF218 proteins, the structure of YdcF is known, revealing a fold similar to the adenine nucleotide α-hydrolase family. YdcF has been shown to bind S-adenosylmethionine and predicted to be an enzyme involved in anaerobic metabolism [26]. Additionally, the conserved amino acids in the DUF218 domain family hint these family members might have a potential enzymatic activity. In support of this, three residues that form a putative catalytic triad have been shown to be important for SanA function. It is interesting to speculate that both SanA and ElyC might function as enzymes working on key intermediates in the PG and ECA biosynthetic pathways, respectively.

### Role of divisome proteins in septal PG synthesis

The observation that absence of *sanA* is a broad and general suppressor of several cell division mutants is intriguing. As *sanA* shows increased mDAP incorporation into the sacculus, an attractive possibility is that the basis of suppression is due to enhanced PG synthesis alleviating the division defects in these mutants. Bacterial cell division is driven by divisome, a multi-protein complex. In the early stages of division, FtsZ, along with ZipA, FtsA and other associated factors, establishes the FtsZ-ring at the division site. Subsequently, a series of Fts proteins (FtsE/X, FtsK, FtsQ, FtsL, FtsB, FtsW, FtsI, FtsN, FtsP) are recruited to facilitate the process of cell division. Of these, several proteins are known to be involved in septal PG synthesis [5,15,29,30], however, the precise molecular function of few of these proteins is not completely understood. *sanA* deletion suppressing the division phenotypes of mutants defective in *ftsE*, -*X*, -*K*, -*Q* and -*P* provides additional evidence that these factors are directly/indirectly involved in septal PG synthesis. In contrast, *sanA* deletion did not suppress *ftsZ84* mutation, but rather exacerbated the phenotype (Fig 2F) indicating optimal levels of FtsZ could be required to establish the Z-ring as the PG sacculus in this mutant could be dense/ aberrant; however, further experiments are required to validate this idea.

## Materials and methods

### Media and growth conditions

Strains were grown in LB (1% tryptone, 0.5% yeast extract, and 1% NaCl) unless otherwise indicated. LBON is LB without NaCl. Nutrient broth (NB) comprises 0.3% beef extract and 0.5% peptone. 1.5% agar is added to the respective broths to make solid media. Minimal A medium (Min-A) was supplemented with 0.005% vitamin B1, 1mM MgSO4, and 0.2% glucose before use [31]. Antibiotics were used at the following concentrations unless specified: Ampicillin (Amp-50 μg/mL), Kanamycin (Kan-50 μg/mL), and Chloramphenicol (Cm-25 μg/mL). Isopropyl β, D-thiogalactopyranoside (IPTG) was used at specified concentrations. Cells were grown at 37°C unless otherwise specified. Growth was monitored by measuring optical density at 600 nm (OD_600_).

### Strains and plasmid constructions

All the strains, plasmids, and primers used in this study are detailed in Supplementary Information (S1 Text).

### Viability assays

Viability assays were performed by serially diluting the overnight culture (10^-2^, 10^-4^, 10^-5^, and 10^-6^), placing 4 μL of each dilution on indicated plates, and growing at a specified temperature for 18–24 h. Permeability assays were done on LB plates supplemented with 1% SDS + 0.5 mM EDTA or Vancomycin (200 μg/mL).

### Microscopy

Overnight cultures were sub-cultured with 1:100 dilution and grown until required OD_600_. At this point, cells were collected, washed, immobilized onto a 1% agarose pad, and visualized under the Zeiss apotome microscope in differential interference contrast mode (DIC, Normasky optics). For cell viability, Live/Dead backlight bacterial viability kit (Invitrogen) was used and cells were visualized by fluorescence microscopy with GFP and Texas red filters.

### Molecular and genetic techniques

Plasmid and recombinant DNA constructions are described in S1 Text. Genomic DNA from MG1655 was used as a template for PCR amplification. All plasmids and strains were confirmed through sequencing and phenotypes. Transformations and P1 phage-mediated transductions were performed as previously described [31]. All strains are derivates of MG1655 unless otherwise mentioned. Deletion mutations are sourced from the Keio collection and transferred to the required strain background through P1-mediated phage transductions. The antibiotic resistance marker was flipped using pCP20.

### Estimation of nascent PG synthesis by mDAP incorporation assay

The ^3^H-mDAP (tritiated meso-diaminopimelic acid) incorporation assay was performed as described earlier [23]. Indicated strains lacking LysA (to prevent the formation of lysine from mDAP) were grown overnight in LB broth. The next day, cells are washed and sub-cultured at 1:100 dilution in Minimal medium supplemented with 0.2% glucose and 0.5% CAA. At OD_600_ of 0.4-0.6, normalized fractions are collected and incubated with 5 μCi/mL of ^3^H-mDAP (Moravek Biochemicals, USA) for 10 min with gentle shaking at 37°C. Cells were immediately lysed by addition of 3 mL of 4% SDS and boiled for 1h. The mixture was cooled overnight at RT and filtered through a 0.22 μm filter. The insoluble PG sacculi collected on the filters were washed with 30 mL of Milli-Q water and dried. The filters were used for counting radioactivity in a liquid scintillation counter (Perkin-Elmer). All the measurements were done at least three times and ratios were calculated and plotted as bar graphs.

### Dot blot assay

Indicated strains were grown overnight in LB at 37°C. The next day, cells were sub-cultured with 1:100 dilution and were grown at 43°C till OD_600_ value of 3. Cells were normalized to 5 OD, pelleted, washed with PBS once and resuspended in 100 µL of PBS containing 10 mM EDTA. The cell suspension was sonicated for 1 min with 10 sec on and 10 sec off cycles at 30% amplitude and briefly spun to remove un-lysed cells and debris. To normalize the sample, protein estimation was done using a Pierce BCA estimation kit (Thermo-Fisher Scientific). Normalised samples were serially diluted (10^-1^, 10^-2^) and 4 µL of each dilution were spotted onto the nitrocellulose membrane. Blots were developed using enhanced chemiluminescence prime-detection substrate (Amersham). Primary anti-ECA antibody was used (a kind gift from Jilong Qin and Renato Morona) at a dilution of 1:1000 and anti-FtsZ antibody was used at 1:50,000. HRP-conjugated anti-rabbit secondary antibody was used at 1:10,000.

### Isolation of suppressors of *ftsI23* mutant using random transposon insertion mutagenesis

To isolate suppressors of *ftsI23* mutant, we performed random insertion mutagenesis using λ1098 containing Tn*10*dTet transposon as described [31]. Briefly, overnight culture of the MG1655 strain carrying the *ftsI23* allele was sub-cultured in LB with 0.4% maltose and 10 mM MgSO4 and grown till the late logarithmic phase at 30°C. Transpositions were done as described [31] and colonies were selected on LB agar supplemented with Tetracycline (Tet; 10 μg/mL) at 32°C. Colonies that grew very well were purified and their suppressive ability was checked by reintroducing the Tn*10*dTet mutations into the *ftsI23* strain using P1 phage-mediated transduction. Once confirmed, the Tet element from the genomic DNA of the suppressor was cloned into a plasmid vector and sequenced using Tet-specific outward primers, P1: 5’-TGGTCACCAACGCTTTTCCCGAG-3’ and P2: 5’-CTGTTGACAAAGGGAATCATAG -3’. By reading the junction sequences, we were able to identify that the insertion was in *sanA* ORF. The *sanA*::Tn*10*dTet insertion mutation exactly behaved like that of Δ*sanA*::Kan deletion from Keio collection and we subsequently used the Keio deletion mutation for all the experiments reported in this study.

### Identification of multicopy plasmid suppressors of SanA^−^ phenotype

We utilized a multicopy plasmid library carrying overlapping *E. coli* (MG1655) genomic DNA fragments of approximately 3–5 kb in size cloned at the BamH1 site in a p15A-based plasmid, pACYC184 (obtained from Miroslav Radman’s laboratory) to perform genetic screens to identify the suppressors of either ∆*sanA* or ∆*sanA wecA* mutants. We transformed the plasmid library into the indicated strains and selected colonies that grew better on NA-Cm plates at 43°C for the ∆*sanA* mutants or 37°C for the ∆*sanA wecA* double mutants. Plasmids were isolated from these colonies and their suppressive ability was confirmed by a subsequent round of transformation and phenotype testing. Finally, the plasmids were sequenced using TetA (5′-CGCCGAAACAAGCGCTCATGAGCC-3’) and TetB primers (5′-CTATGCGCACCCGTTCTCGGAGCAC-3’) to identify the region responsible for the suppression.

### Statistical analysis

All experiments were conducted at least three times. Error bars in the graphs are depicted as mean ± SD. We employed an unpaired student T-test to compare the significance between the two samples. In all the graphs, *, *P* < 0.05; **, *P* < 0.005; ***, *P* < 0.001 and ****, *P* < 0.0001.

## Supporting information

S1 Text Details of strain and plasmid constructions, supplemental methods, protocols and references are given. Tables A and B in S1 Text describe list of strains and plasmids used in this study.
(DOCX)

S1 Fig Effect of *sanA* deletion on growth of cell division mutants. (A) Indicated strains were grown overnight at 30°C in LB, serially diluted and viability was assessed by spotting 4 μL of each dilution on LB plates by incubation at 30°C and 37°C. (B) Indicated strains were grown in LB at 30°C and cell viability was assessed as described above on LB + 1% NaCl and LBON 30°C (for *ftsX*), LB 30°C and LBON 42°C (for *ftsQ1*), LB 37°C and LBON 42°C (for ∆*ftsP*). (C) Viability of indicated strains (for *ftsA12*) was tested on LB at 30°C and 42°C. (D) Viability of WT and indicated mutant strains (∆*amiABC*) was assessed on LB and NA at 37°C.
(TIF)

S2 Fig Effect of SanA on envelope processes. (A,B) Growth of indicated strains was examined on LB or NA plates at 37°C. (C) HPLC chromatograms showing PG composition of WT and ∆*sanA* mutant strains. PG sacculi were isolated and analysed by Reverse Phase-HPLC (RP-HPLC) as described in the SI-methods. (D) Table depicting the identity of the muropeptide peaks and their area % calculations. Peaks 6–12 (highlighted) were considered in calculating the total cross-linking percentage. Values represent the mean ± standard deviation.
(TIF)

S3 Fig SanA^−^ phenotypes. (A) Viability assay showing the osmoremedial phenotype of ∆*sanA* mutant. Growth of WT and *sanA* mutant on LB, NA or NA supplemented with 10% sorbitol or 0.2M NaCl at 43°C. (B) Estimation of LPS in WT and ∆*sanA* mutant. Normalized cultures were processed as described in SI-methods for LPS and FtsZ visualization. C1 and C2 represent two biological replicates (C) Cell morphology of WT and ∆*sanA* mutant. Cells were grown in LB, NB or Min-A at 37°C till OD_600_ of 0.6-0.8 and visualized using DIC microscopy. The scale bar represents 5 μm. For cell length measurements, approximately 100 cells were used and data were analysed by ImageJ software.
(TIF)

S4 Fig Multicopy suppressors of SanA^−^ phenotypes. (A,B) Viability assays of indicated strains on LB, NA, LB + 200 µg/mL vancomycin or LB + 1.0% SDS + 0.5 mM EDTA at 43°C. C2 clone was obtained in *sanA* mutant as a multicopy suppressor from pACYC184 plasmid library whereas W14 and W15 were obtained in *sanA wecA* double mutant. C2 and W14 clones contain the region encompassing the *wecA* and *wzzE* genes whereas W15 has *grxD-mepH* region.
(TIF)

S5 Fig Comparative structural analysis of SanA, ElyC, and YgjQ. (A) AlphaFold-predicted structures of SanA, ElyC, and YgjQ, highlighting the conserved threonine, histidine, and glutamate residues that form a putative catalytic triad. (B) Western blot analysis of plasmid borne SanA-His and its site-directed mutant variants. Indicated strains were cultured in LB + Amp + 10 μM IPTG, and normalized cell fractions were subjected to western blotting to assess their expression levels. FtsZ is used as a loading control.
(TIF)
