# Supplementary material for: Identification of SanA as a novel regulator of peptidoglycan biogenesis in Escherichia coli
Source: PLoS Genet. 2025 May 22;21(5):e1011712. doi: 10.1371/journal.pgen.1011712 (PMC12176290; doi:10.1371/journal.pgen.1011712)
Supplement: S1 Text — Tables A and B in S1 Text describe list of strains and plasmids used in this study. (DOCX) [file pgen.1011712.s001.docx]

**Supplementary Information:**

**Identification of SanA as a novel regulator of peptidoglycan biogenesis in *Escherichia coli***

Bhargavi Gundavarapu^1,2,¶^, Krishna Chaitanya Nallamotu^1,2,¶^, Vishnu Vachana Murapaka^1,2^, Balaji Venkataraman^1^, L Saisree^1^, Manjula Reddy^1,2,*^

^1^CSIR-Centre for Cellular and Molecular Biology, Hyderabad, India

^2^Academy of Scientific and Innovative Research (AcSIR), Ghaziabad, India

^¶^Equal contribution

^*^manjula@ccmb.res.in, mreddy65@gmail.com

**Supplementary Materials and Methods:**

**Plasmid constructions:**

For PCR amplifications, the genomic DNA of the MG1655 strain was used as a template. DNA was amplified using Q5 High-Fidelity DNA polymerase (NEB), and the clones obtained were confirmed by sequence analysis.

**pKMR1:** The *sanA* gene, along with its ribosome binding site (RBS), was PCR amplified (with restriction sites underlined) using forward and reverse primers:

5’-GCTCTAGACCTTAGATCGAGTCTCCTGCATG-3’ and 5’-CCCAAGCTTCTAGTGATGGTGATGGTGATGCTTTCCTTGTTTCTTTTGTAATTC-3’ respectively, and the resulting DNA fragment was cloned at Xbal-HindIII sites of pTrc99a plasmid to generate a C-terminal 6XHis fusion construct. This clone complemented *sanA* mutant phenotype at 10 μM IPTG.

**pKMR3:** The region encompassing the *wecA-wzzE* genes was PCR amplified using the forward and reverse primers:

5’-CGGAATTCTTTCTCTGAGAGCATGCATTGTGAATTTACTGACAGTG-3’

5’-GCTCTAGACTATTTCGAGCAACGGCGGGTTAATGCG-3’ respectively, and the resulting DNA fragment was cloned at EcoRI-XbaI sites of pTrc99a plasmid.

**pKMR4:**  This plasmid is a pTrc99 derivative carrying *^ss^dsbA-sanA* fusion. Here, the N-terminal transmembrane domain (amino acids 1-24) of SanA was replaced with a DsbA signal sequence (*^ss^dsbA*) consisting of 19 amino acids, which now targets the protein to soluble periplasmic space (1). Additionally, a 6x His tag was added to its C-terminus. This construct was cloned into the pTrc99a vector at the XbaI and HindIII sites using the following forward and reverse primers:

5’-GCTCTAGAATGAAAAAGATTTGGCTGGCGCTGGCTGGTTTAGTTTTAGCGTTTAGCGCATCGGCGATGGCGGATCGCTGGATGAGCTGGAAAAC-3'

5’-CCCAAGCTTCTAGTGATGGTGATGGTGATGCTTTCCTTGTTTCTTTTGTAATTC-3'

**pKMR5:** To generate a site-directed variant of the *sanA* gene (Threonine^125^ mutated to Alanine), a three-step PCR was performed as described in the methods with the following primers:

Forward and reverse SDM primers:

FP - 5’-GATTACGCAGGCTTTCGTGCGCTGGATTCCATCGTGCGTAC-3’

RP - 5’-GTACGCACGATGGAATCCAGCGCACGAAAGCCTGCGTAATC-3’

Flanking primers:

FP - 5’-GCTCTAGACCTTAGATCGAGTCTCCTGCATG-3’

RP - 5’-CCCAAGCTTCTAGTGATGGTGATGGTGATGCTTTCCTTGTTTCTTTTGTAATTC-3’ respectively and the resulting amplicon was cloned at Xbal-HindIII sites of pTrc99a plasmid to generate a C-terminal 6XHis fusion construct.

**pKMR6:** To generate a site-directed variant of the *sanA* gene (Histidine^149^ mutated to Alanine), a three-step PCR was performed as described in the methods with the following primers:

Forward and reverse SDM primers:

FP - 5’-CATTATTATCACCCAACGTTTCGCGTGTGAGCGAGCATTATTTATTG-3’

RP - 5’-CAATAAATAATGCTCGCTCACACGCGAAACGTTGGGTGATAATAATG-3’

Flanking primers:

FP - 5’-GCTCTAGACCTTAGATCGAGTCTCCTGCATG-3’

RP - 5’-CCCAAGCTTCTAGTGATGGTGATGGTGATGCTTTCCTTGTTTCTTTTGTAATTC-3’ respectively and the resulting amplicon was cloned at Xbal-HindIII sites of pTrc99a plasmid to generate a C-terminal 6XHis fusion construct.

**pKMR7:** To generate a site-directed variant of the *sanA* gene (Cysteine^150^ mutated to Alanine), a three-step PCR was performed as described in the methods with the following primers:

Forward and reverse SDM primers:

FP - 5’-CACCCAACGTTTCCACGCGGAGCGAGCATTATTTATTGCGC-3’

RP - 5’-GCGCAATAAATAATGCTCGCTCCGCGTGGAAACGTTGGGTG-3’

Flanking primers:

FP - 5’-GCTCTAGACCTTAGATCGAGTCTCCTGCATG-3’

RP - 5’-CCCAAGCTTCTAGTGATGGTGATGGTGATGCTTTCCTTGTTTCTTTTGTAATTC-3’ respectively and the resulting amplicon was cloned at Xbal-HindIII sites of pTrc99a plasmid to generate a C-terminal 6XHis fusion construct.

**pKMR8:** To generate a site-directed variant of the *sanA* gene (Glutamate^182^ mutated to Alanine), a three-step PCR was performed as described in the methods with the following primers:

Forward and reverse SDM primers:

FP - 5’-GCTGTCAGTACGTATTCGTGCGTTTGCCGCCCGTTTCGGTGC-3’

RP - 5’-GCACCGAAACGGGCGGCAAACGCACGAATACGTACTGACAGC-3’

Flanking primers:

FP - 5’-GCTCTAGACCTTAGATCGAGTCTCCTGCATG-3’

RP - 5’-CCCAAGCTTCTAGTGATGGTGATGGTGATGCTTTCCTTGTTTCTTTTGTAATTC-3’ respectively and the resulting amplicon was cloned at Xbal-HindIII sites of pTrc99a plasmid to generate a C-terminal 6XHis fusion construct.

**Supplementary methods:**

**Peptidoglycan (PG) isolation**

PG sacculi isolation was done as described earlier with slight modifications (2,3). Indicated strains were grown overnight in LB at 37°C. The next day, cells were grown to the desired OD_600_ value and harvested by centrifugation at 6000 x g for 10 min. The cell pellet was resuspended in ice-cold deionised water and added dropwise to boiling 8% SDS solution. This suspension was boiled for 50 min and left overnight at RT to cool down. The suspension was then diluted with deionised water and centrifuged at 300000 x g for 50 min in an ultracentrifuge. The obtained crude PG pellet was washed multiple times with water until all the SDS were removed. Further, the PG pellet was treated with α-amylase (100 μg/mL in 10mM Tris-HCL, pH 7.2 for 2 h at 37°C), followed by heat-activated pronase (200 μg/mL for 90 min at 60°C) to remove glycogen and protein components associated with the sacculi. 8% SDS was added to inactivate these enzymes, and the suspension was boiled for 10 min. The suspension was then centrifuged to collect PG sacculi, and the supernatant was discarded. The PG sacculi were washed repeatedly with water to remove SDS and subsequently resuspended in 25 mM Tris-Cl pH 8.0.

**Analysis of PG muropeptides by Reverse Phase-HPLC (RP-HPLC)**

RP-HPLC was performed as described earlier (2,3). PG sacculi were treated with a muramidase, mutanolysin (Sigma-Aldrich) overnight at 37°C to obtain soluble muropeptides. Muropeptides were reduced by addition of 1mg sodium borohydride in 50 mM of sodium borate buffer, pH 9.0 for 30 min at RT. Excess sodium borohydride is destroyed by the addition of 20% orthophosphoric acid and pH is adjusted to 3-4 and the sample is applied to a Zorbax C18 RP-HPLC column. A gradient of 1-10% acetonitrile in water containing 0.1% TFA was used for elution. Absorbance was detected at 205 nm.

**Analysis of Lipopolysaccharide (LPS)**

LPS analysis was done as described previously (4). Cells were grown and harvested at OD_600_ of 1.0. The cell pellets (normalized to the absorbance OD_600_ of 1.0) were mixed with 50 μL of LPS sample buffer (660 mM Tris-HCl, pH 7.6, 10% w/v glycerol, 2% w/v SDS, 4% v/v 2-β-mercaptoethanol, 0.1% w/v bromophenol blue), and boiled for 10 min. 10 μL of proteinase K (2.5 mg/mL in sample buffer) was added to the 50 μL of above boiled sample and incubated at 56°C for 4 h. The samples were centrifuged, and supernatants were diluted further (1:5) in sample buffer. 5 μL of sample was loaded on 20% tricine-SDS polyacrylamide gel and LPS was visualized by silver staining.

**Western blotting analysis**

Cell lysates were made as described earlier (4), samples were run on SDS-PAGE (14%) and transferred onto PVDF membrane using semi-dry western transfer method. After completion of transfer, the membrane was blocked in 5% skimmed milk in 1x TBST for 1h at RT and incubated overnight at 4°C with primary antibody (α- FtsZ at a dilution of 1:50000 or α- His at 1:10000). Later, the blot was washed 5 times with 1x TBST and incubated with anti-rabbit secondary antibody-HRP conjugate (at a dilution of 1:10000) for 1h at RT. Blot was further washed 5 times with 1x TBST and bands were visualized with ECL Prime detection substrate (Amersham) and were developed using Chemi-Doc system.

**Construction of Site-directed mutants**

In the first PCR reaction, a flanking forward primer was used together with a reverse primer containing the desired mutation. In the second reaction, a forward primer carrying the mutation was paired with a flanking reverse primer. These two PCR reactions produced overlapping fragments, each incorporating the desired mutation at one end. In the third and final PCR step, the two overlapping fragments were mixed in a 1:1 molar ratio and used as templates in a reaction with the flanking forward and reverse primers. The resulting amplicon containing the desired mutation was used to clone into the pTrc99a plasmid. The presence of mutation was confirmed through sequencing.

**Strain constructions:**

The gene deletion strains used in this study were sourced from the Keio collection (5). The presence of gene deletions was confirmed by PCR amplification and sequence analysis. The deletions were introduced into desired strains by P1 phage-mediated transductions. The *kan* gene cassette was flipped out wherever required using pCP20 plasmid (6). All strains are MG1655 derivatives unless otherwise indicated.

**Table A. List of strains used in this study**

| **Strain** | **Genotype** | **Source/Reference** |
| --- | --- | --- |
| MG1655 | *rph1 ilvG rfb-50* | Lab collection |
| KMR001 | *ΔsanA*::*kan^R^* | This study |
| KMR002 | *ΔsanA::frt* | This study |
| KMR003 | *ftsI23 leuB82*::Tn*10 tet^R^* | Lab collection |
| KMR004 | *ftsI23* | This study |
| KMR005 | *ftsI23 sanA::Tn10dtet* | This study |
| KMR006 | KMR003 *ΔsanA*::*kan* | This study |
| KMR007 | KMR003/ pTrc99a | This study |
| KMR008 | KMR003/ pKMR1 | This study |
| KMR009 | KMR004/ pTrc99a | This study |
| KMR0010 | KMR004/ pKMR1 | This study |
| KMR0011 | KMR003 *ΔyeiS*::*kan^R^* | This study |
| KMR0012 | *ftsE::*Tn*10*dCm | Lab collection |
| KMR0013 | KMR0012 *ΔsanA*::*kan^R^* | This study |
| KMR0014 | *ftsK44 zbj-1230::Tn10 tet^R^* | Lab collection |
| KMR0015 | KMR0014 *ΔsanA*::*kan^R^* | This study |
| KMR0016 | *ftsZ84 leuB82::Tn10 tet^R^* | Lab collection |
| KMR0017 | KMR0016 *ΔsanA*::*kan^R^* | This study |
| KMR0018 | *ftsX::*Tn*10*dCm | Lab collection |
| KMR0019 | KMR0018 *ΔsanA*::*kan^R^* | This study |
| KMR0020 | *ftsQ1 leuB::Tn10 tet^R^* | Lab collection |
| KMR0021 | KMR0020 *ΔsanA*::*kan^R^* | This study |
| KMR0022 | *ΔftsP::kan^R^* | Lab collection |
| KMR0023 | KMR002 Δ*ftsP::kan^R^* | This study |
| KMR0024 | *lpxC1272 leuB::Tn10 tet^R^* | Lab collection |
| KMR0025 | KMR0024 *ΔsanA*::*kan^R^* | This study |
| KMR0026 | *ΔfabH::kan^R^* | This study |
| KMR0027 | KMR002 *ΔfabH*::*kan^R^* | This study |
| KMR0028 | BW27783 *∆mrcA ∆mrcB /* P_ara_:: *mrcA cm^R^* | Lab collection |
| KMR0029 | KMR0028 *ΔsanA*::*kan^R^* | This study |
| KMR0030 | BW27783 *∆mrcA ∆mrcB /* P_lac_:: *mrcB amp^R^* | Lab collection |
| KMR0031 | KMR0030 *ΔsanA*::*kan^R^* | This study |
| KMR0032 | MG1655 *pbpA45*  Tet^R^ | (7) |
| KMR0033 | KMR0032 *ΔsanA*::*kan^R^* | This study |
| KMR0034 | MG1655 *ΔlysA::kan^R^* | This study |
| KMR0035 | MG1655 *ΔlysA::frt* | This study |
| KMR0036 | KMR0035 *ΔsanA::kan^R^* | This study |
| KMR0037 | KMR0035 *ΔsanA::frt* | This study |
| KMR0038 | KMR0035 / pTrc99a | This study |
| KMR0039 | KMR0035 / pKMR1 | This study |
| KMR0040 | KMR0037 / pTrc99a | This study |
| KMR0041 | KMR0037 / pKMR1 | This study |
| KMR0042 | KMR0035 *ftsI23 leuB82::Tn10 tet^R^* | This study |
| KMR0043 | KMR0037 *ftsI23 leuB82::Tn10 tet^R^* | This study |
| KMR0044 | KMR0035 *ftsE::*Tn*10*dCm | This study |
| KMR0045 | KMR0044 *ΔsanA::kan^R^* | This study |
| KMR0046 | KMR0035 *ftsZ84 leuB82::Tn10 tet^R^* | This study |
| KMR0047 | KMR0037 *ftsZ84 leuB82::Tn10 tet^R^* | This study |
| KMR0048 | KMR0030 *ΔlysA::kan^R^* | This study |
| KMR0049 | KMR0048 *ΔsanA::tet^R^* | This study |
| KMR0050 | KMR0035 *pbpA45.tet^R^* | This study |
| KMR0051 | KMR0050 *ΔsanA::kan^R^* | This study |
| KMR0052 | MG1655 / pTrc99a | This study |
| KMR0053 | MG1655 / pKMR1 | This study |
| KMR0054 | KMR002 / pTrc99a | This study |
| KMR0055 | KMR002 / pKMR1 | This study |
| KMR0056 | MG1655 / pACYC184 | This study |
| KMR0057 | KMR002 / pACYC184 | This study |
| KMR0058 | KMR002 / pACYC184 C2 | This study |
| KMR0059 | KMR002 / pACYC184 W14 | This study |
| KMR0060 | KMR002 / pACYC184 W15 | This study |
| KMR0061 | MG1655 / pKMR3 | This study |
| KMR0062 | KMR002 / pKMR3 | This study |
| KMR0063 | KMR0034 / pKMR3 | This study |
| KMR0064 | KMR0036 / pKMR3 | This study |
| KMR0071 | *ΔelyC*::*kan^R^* | This study |
| KMR0072 | KMR002 *ΔelyC*::*kan^R^* | This study |
| KMR0073 | KMR0035 *ΔelyC::kan^R^* | This study |
| KMR0074 | KMR0037 Δe*lyC*::*kan^R^* | This study |
| KMR0075 | *ΔwecA::Kan^R^* | This study |
| KMR0076 | *ΔwecG::Kan^R^* | This study |
| KMR0077 | *ΔwecF::Kan^R^* | This study |
| KMR0078 | *ΔwzxE::Kan^R^* | This study |
| KMR0079 | *ΔwzzE::Kan^R^* | This study |
| KMR0080 | KMR002 *ΔwecA*::*Kan^R^* | This study |
| KMR0081 | KMR002 *ΔwecG*::*Kan^R^* | This study |
| KMR0082 | KMR002 *ΔwecF*::*Kan^R^* | This study |
| KMR0083 | KMR002 *ΔwzxE*::*Kan^R^* | This study |
| KMR0084 | KMR002 *ΔwzzE*::*Kan^R^* | This study |
| KMR0085 | KMR0080 / pTrc99a | This study |
| KMR0086 | KMR0080 / pMN82 | This study |
| KMR0087 | KMR002 / pMN82 | This study |
| KMR0088 | KMR002 / pMN81 | This study |
| KMR0089 | KMR002 / pMN80 | This study |
| KMR0090 | KMR002 / pKMR4 | This study |
| KMR0091 | Δ*mepS*::*frt* | This study |
| KMR0092 | KMR0091 / pTrc99a | This study |
| KMR0093 | KMR0091 / pMN82 | This study |
| KMR0094 | KMR0091 / pMN81 | This study |
| KMR0095 | KMR0091 / pMN80 | This study |
| KMR0096 | *ftsA12 leuB82::Tn10 tet^R^* | Lab collection |
| KMR0097 | KMR0096 Δ*sanA*::*Kan^R^* | This study |
| KMR0098 | Δ*amiABC*::*frt* | Lab collection |
| KMR0099 | KMR0098 Δ*sanA*::*Kan^R^* | This study |

**Table B. List of plasmids used in this study**

| **Plasmids** | **Features** | **Source/reference** |
| --- | --- | --- |
| pTrc99a | ColE1, Amp^R^, *lacI^q^*, P_trc_ | Lab collection |
| pCP20 | pSC101(Ts), Amp^R^, Cm^R^, Flp | (6) |
| pACYC184 | p15A, Tet^R^, Cm^R^ | Lab collection |
| pACYC184 C2 and W14 | pACYC184 derivative with *wecA-wzzE* region | Obtained as multicopy suppressor (Fig. S4) |
| pACYC184 W15 | pACYC184 derivative with *grxD-mepH* region | Obtained as a multicopy suppressor (Fig. S4) |
| pKMR1 | pTrc99a-*sanA*-6x His | This study |
| pKMR3 | pTrc99a-*wecA-wzzE* | This study |
| pMN82 | pTrc99a-*mepH (ydhO)* | (3) |
| pMN81 | pTrc99a-*mepM (yebA)* | (3) |
| pMN80 | pTrc99a-*mepS (spr)* | (3) |
| pKMR4 | pTrc99a- ^ss^*dsbA*-*sanA* | This study |
| pKMR5 | pTrc99a-*sanA^T125A^*-6x His | This study |
| pKMR6 | pTrc99a-*sanA^H149A^*-6x His | This study |
| pKMR7 | pTrc99a-*sanA^C150A^*-6x His | This study |
| pKMR8 | pTrc99a-*sanA^E182A^*-6x His | This study |

**Supplemental references:**

1. Schierle CF, Berkmen M, Huber D, Kumamoto C, Boyd D, Beckwith J. (2003) The DsbA Signal Sequence Directs Efficient, Cotranslational Export of Passenger Proteins to the *Escherichia coli* Periplasm via the Signal Recognition Particle Pathway. J Bacteriol 185(19): 5706-5713.
2. Glauner B, Holtje JV, Schwarz U. (1988) The composition of the murein of *Escherichia coli*. J Biol Chem 263: 10088-10095.
3. Singh, S. K., SaiSree, L., Amrutha, R. N., Reddy, M. (2012). Three redundant murein endopeptidases catalyse an essential cleavage step in peptidoglycan synthesis of *Escherichia coli* K12. Molecular Microbiology 86(5):1036-1051.
4. Mahalakshmi, S., Sunayana, M. R., SaiSree, L., Reddy, M. (2013). *yciM* is an essential gene required for regulation of lipopolysaccharide synthesis in *Escherichia coli*. Molecular Microbiology 91(1):145-157.
5. Baba T, Ara T, Hasegawa M, Takai Y, Okumura Y, Baba M, Datsenko KA, Tomita M, Wanner BL, Mori H. (2006). Construction of *Escherichia coli* K-12 in-frame, single-gene knockout mutants: the Keio collection. Molecular Systems Biology 2:2006.0008.
6. Datsenko KA, Wanner BL. (2000). One-step inactivation of chromosomal genes in *Escherichia coli* K-12 using PCR products. Proceedings of the National Academy of Sciences 97:6640–6645.
7. Ogura T, Bouloc P, Niki H, D’Ari R, Hiraga S, Jaffé A. (1989). Penicillin-binding protein 2 is essential in wild-type *Escherichia coli* but not in *lov* or *cya* mutants. J Bacteriol 171(6):3025–30.
