# Supplementary figures and images for: Identification of SanA as a novel regulator of peptidoglycan biogenesis in Escherichia coli
Source: PLoS Genet. 2025 May 22;21(5):e1011712. doi: 10.1371/journal.pgen.1011712 (PMC12176290; doi:10.1371/journal.pgen.1011712)

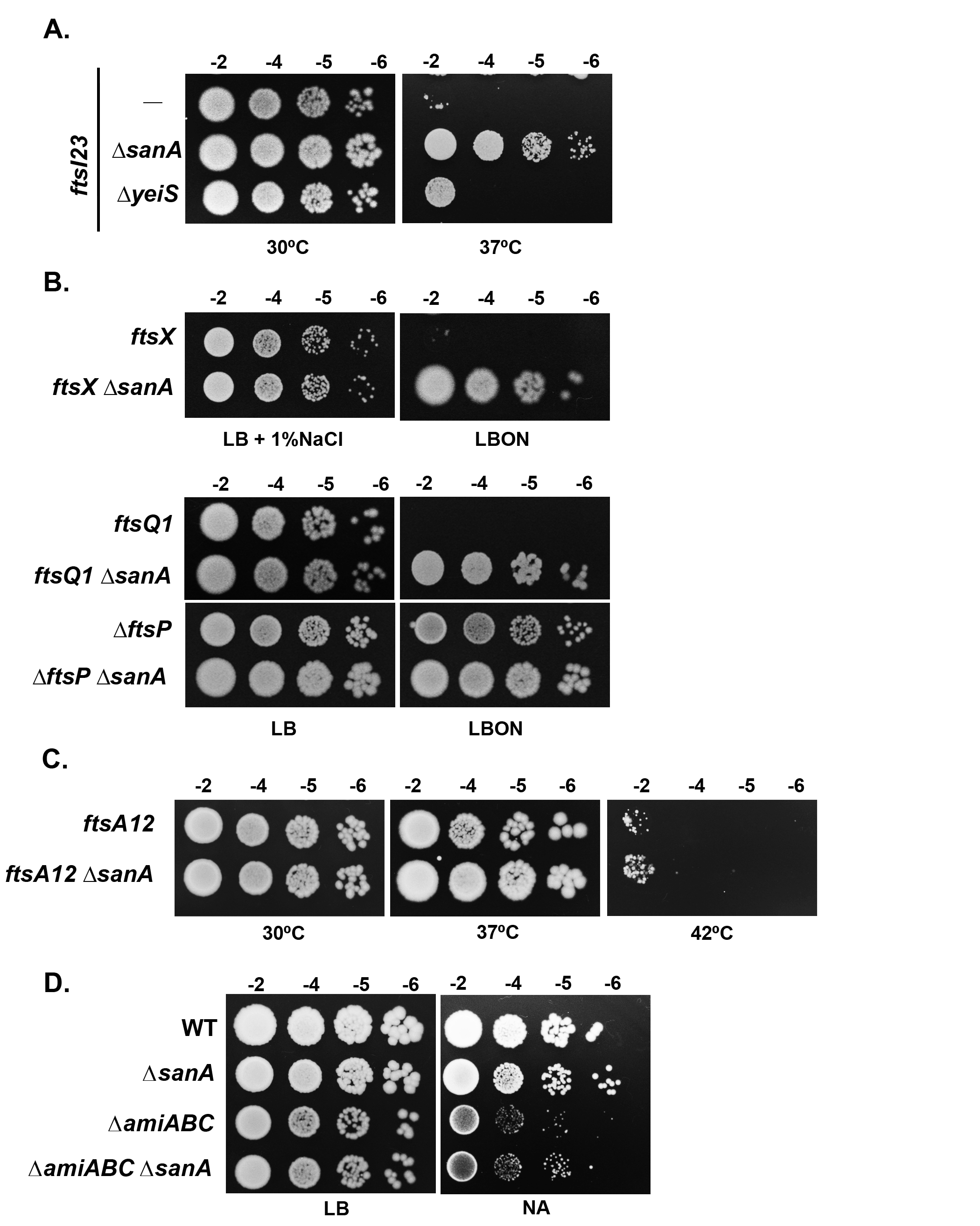

Supplement: S1 Fig — (A) Indicated strains were grown overnight at 30°C in LB, serially diluted and viability was assessed by spotting 4 μL of each dilution on LB plates by incubation at 30°C and 37°C. (B) Indicated strains were grown in LB at 30°C and cell viability was assessed as described above on LB + 1% NaCl and LBON 30°C (for ftsX), LB 30°C and LBON 42°C (for ftsQ1), LB 37°C and LBON 42°C (for ∆ftsP). (C) Viability of indicated strains (for ftsA12) was tested on LB at 30°C and 42°C. (D) Viability of WT and indicated mutant strains (∆amiABC) was assessed on LB and NA at 37°C. (TIF) [file pgen.1011712.s002.tif]

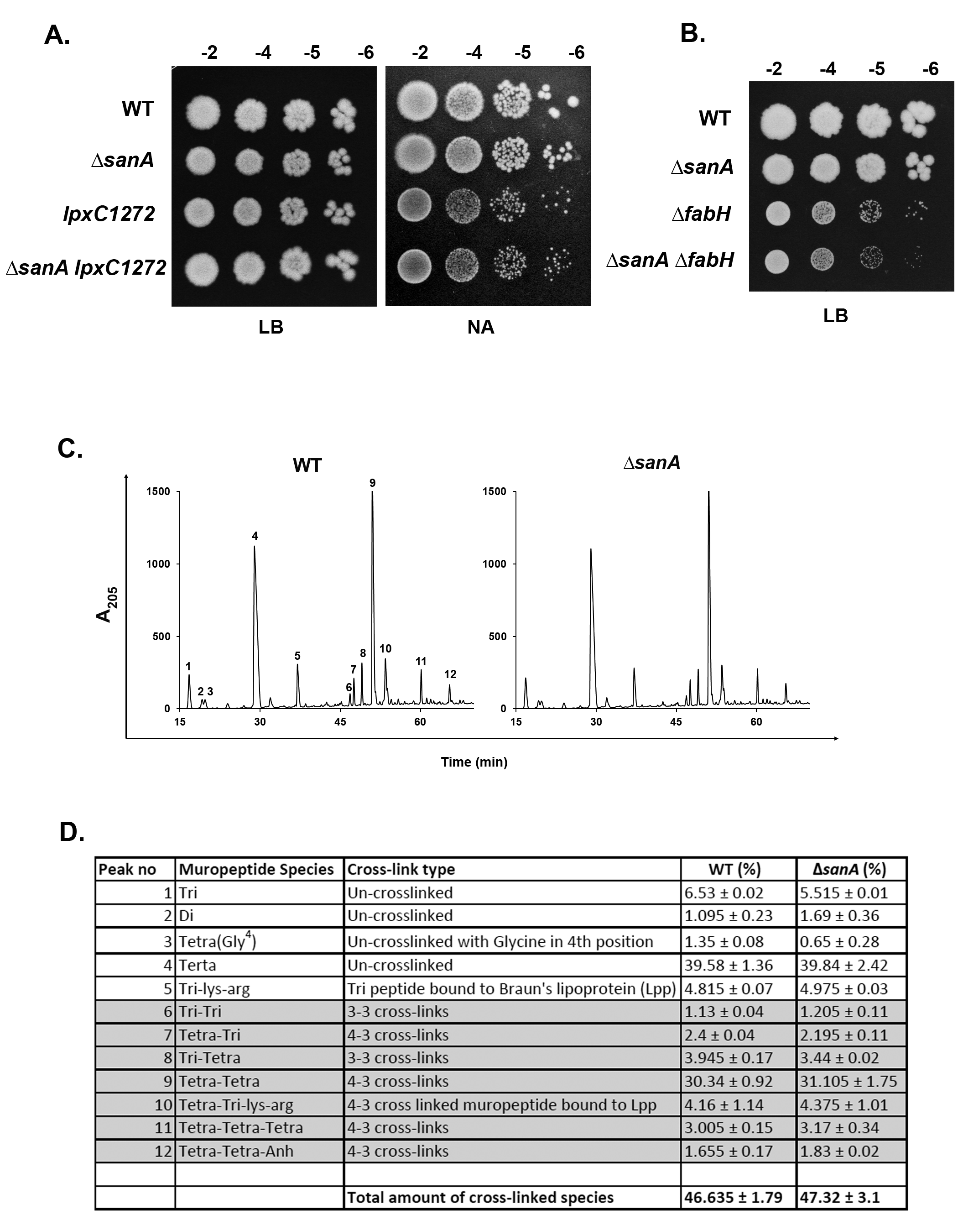

Supplement: S2 Fig — (A,B) Growth of indicated strains was examined on LB or NA plates at 37°C. (C) HPLC chromatograms showing PG composition of WT and ∆sanA mutant strains. PG sacculi were isolated and analysed by Reverse Phase-HPLC (RP-HPLC) as described in the SI-methods. (D) Table depicting the identity of the muropeptide peaks and their area % calculations. Peaks 6–12 (highlighted) were considered in calculating the total cross-linking percentage. Values represent the mean ± standard deviation. (TIF) [file pgen.1011712.s003.tif]

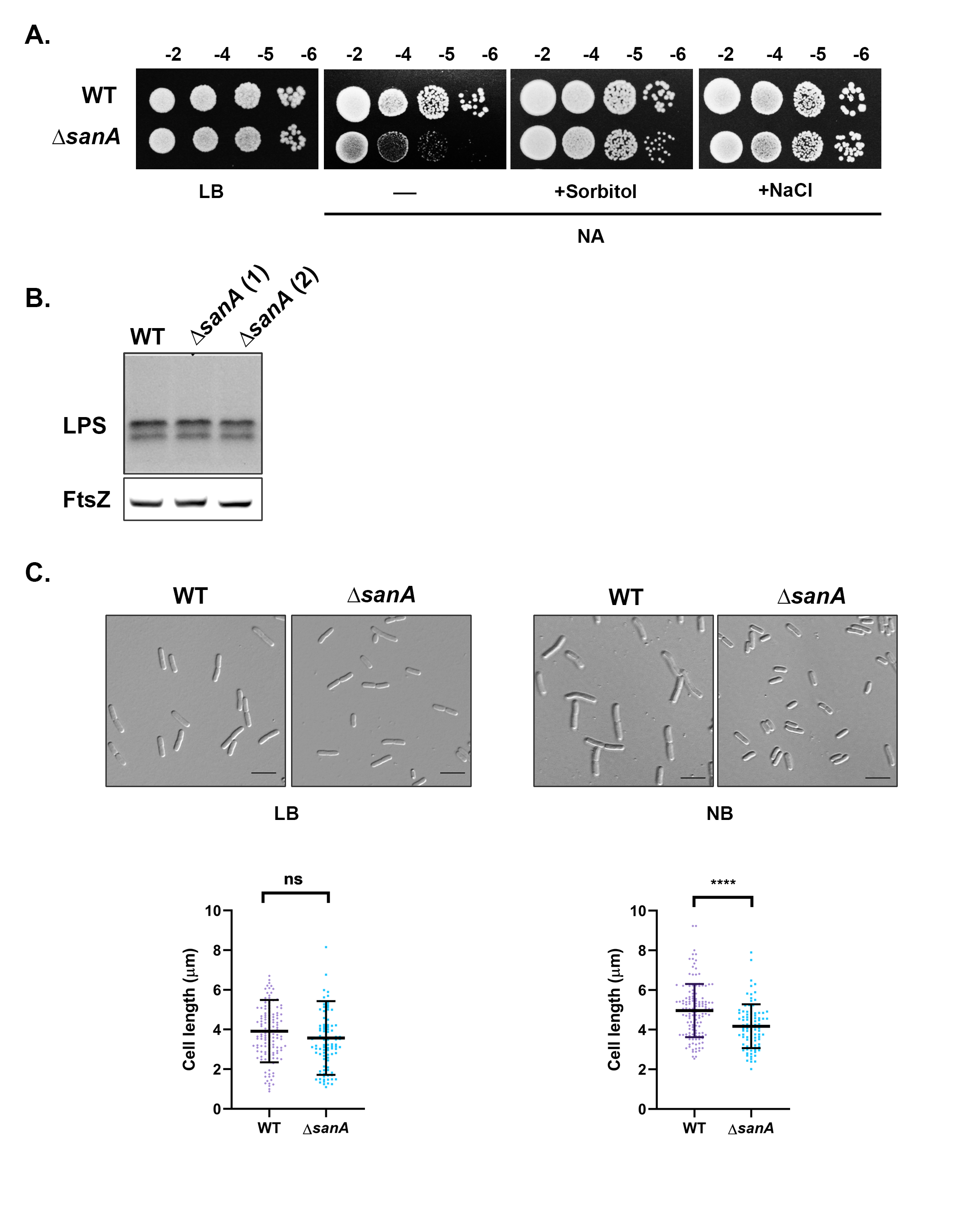

Supplement: S3 Fig — (A) Viability assay showing the osmoremedial phenotype of ∆sanA mutant. Growth of WT and sanA mutant on LB, NA or NA supplemented with 10% sorbitol or 0.2M NaCl at 43°C. (B) Estimation of LPS in WT and ∆sanA mutant. Normalized cultures were processed as described in SI-methods for LPS and FtsZ visualization. C1 and C2 represent two biological replicates (C) Cell morphology of WT and ∆sanA mutant. Cells were grown in LB, NB or Min-A at 37°C till OD600 of 0.6-0.8 and visualized using DIC microscopy. The scale bar represents 5 μm. For cell length measurements, approximately 100 cells were used and data were analysed by ImageJ software. (TIF) [file pgen.1011712.s004.tif]

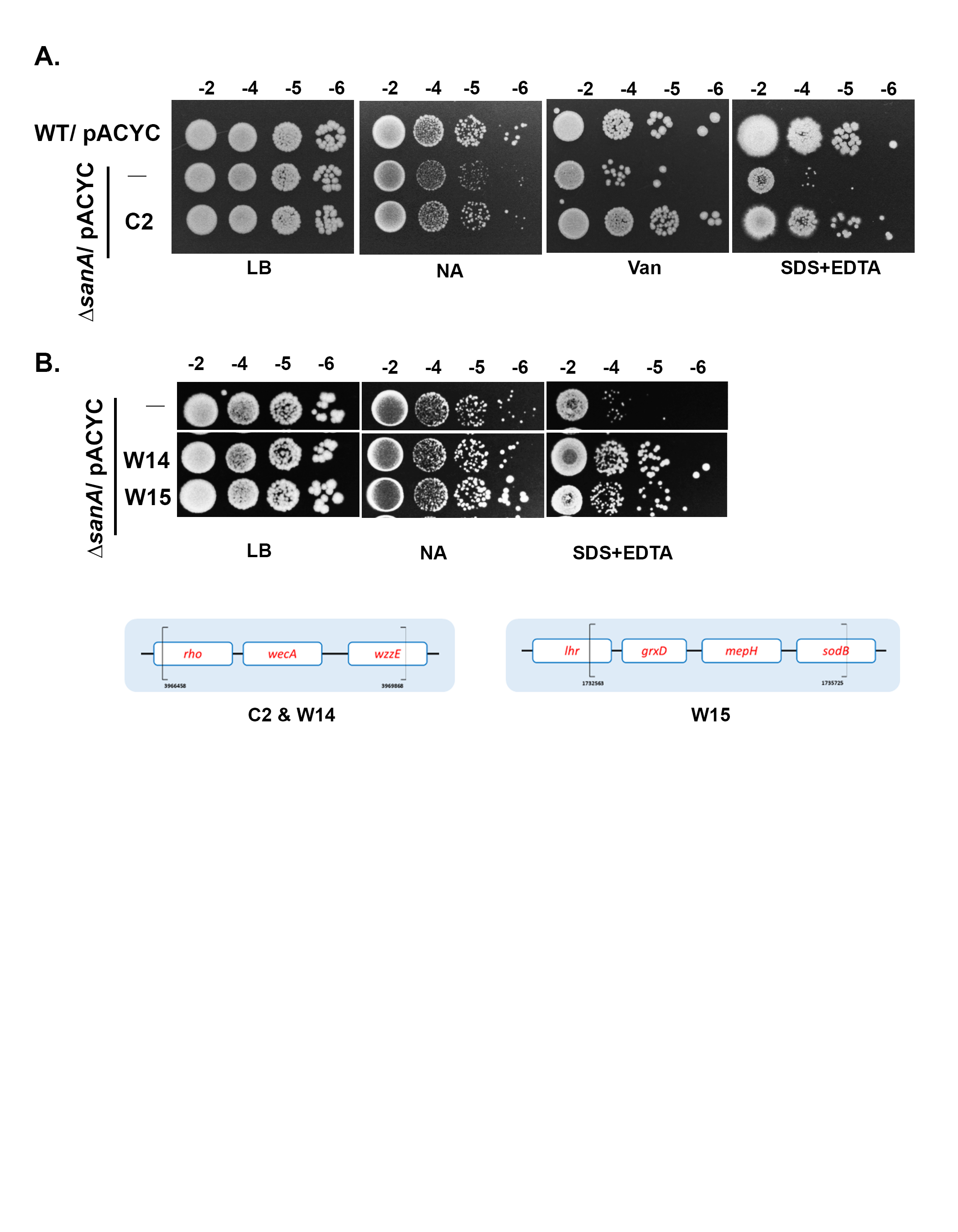

Supplement: S4 Fig — (A,B) Viability assays of indicated strains on LB, NA, LB + 200 µg/mL vancomycin or LB + 1.0% SDS + 0.5 mM EDTA at 43°C. C2 clone was obtained in sanA mutant as a multicopy suppressor from pACYC184 plasmid library whereas W14 and W15 were obtained in sanA wecA double mutant. C2 and W14 clones contain the region encompassing the wecA and wzzE genes whereas W15 has grxD-mepH region. (TIF) [file pgen.1011712.s005.tif]

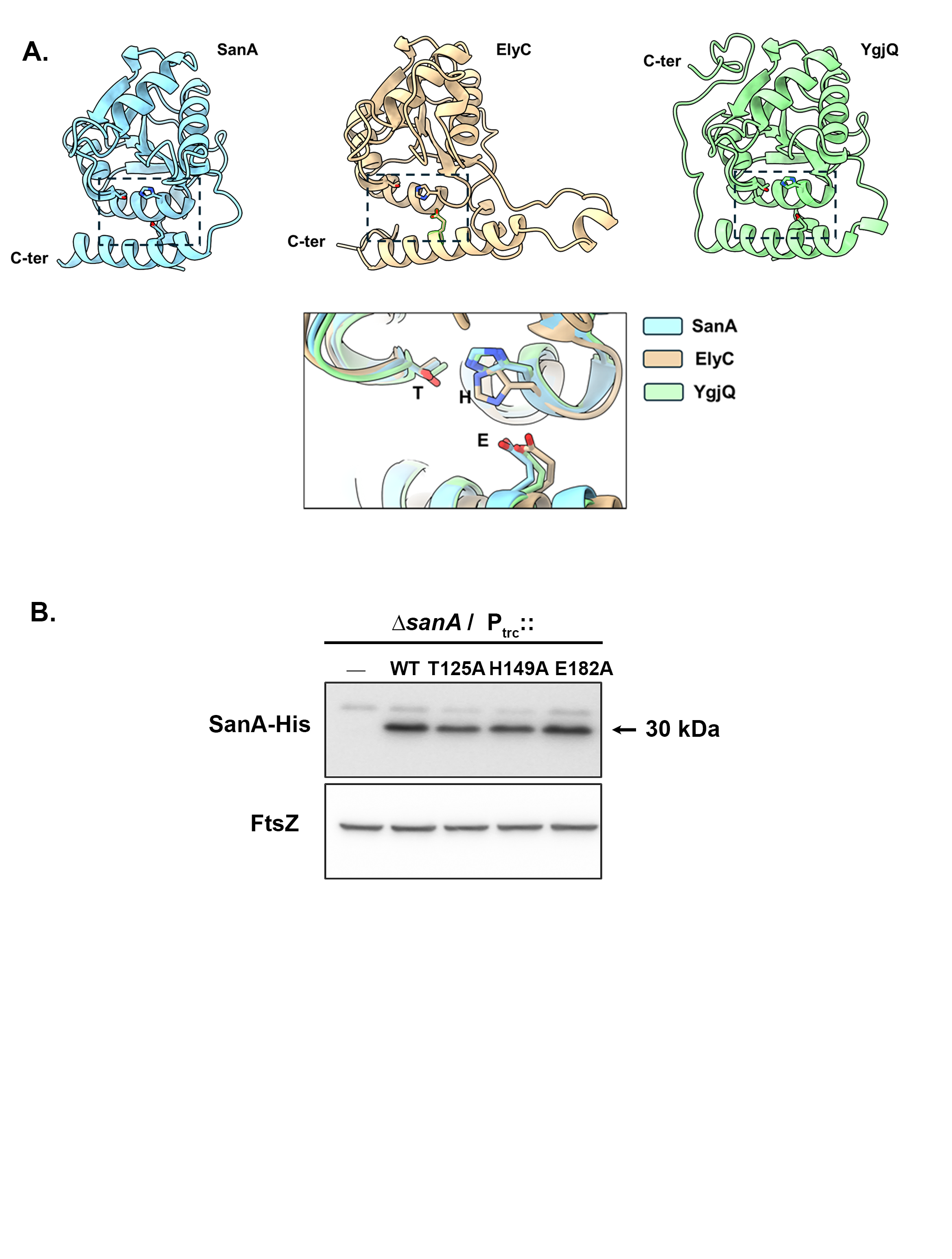

Supplement: S5 Fig — (A) AlphaFold-predicted structures of SanA, ElyC, and YgjQ, highlighting the conserved threonine, histidine, and glutamate residues that form a putative catalytic triad. (B) Western blot analysis of plasmid borne SanA-His and its site-directed mutant variants. Indicated strains were cultured in LB + Amp + 10 μM IPTG, and normalized cell fractions were subjected to western blotting to assess their expression levels. FtsZ is used as a loading control. (TIF) [file pgen.1011712.s006.tif]
